# Comparative Genomic Reveals Clonal Heterogeneity in Persistent *Staphylococcus aureus* Infection

**DOI:** 10.3389/fcimb.2022.817841

**Published:** 2022-02-21

**Authors:** Sabrina Klein, Benedict Morath, Daniel Weitz, Patrick A. Schweizer, Aline Sähr, Klaus Heeg, Sébastien Boutin, Dennis Nurjadi

**Affiliations:** ^1^ Department of Infectious Diseases, Medical Microbiology and Hygiene, Heidelberg University Hospital, Heidelberg, Germany; ^2^ Hospital Pharmacy, Heidelberg University Hospital, Heidelberg, Germany; ^3^ Cooperation Unit Clinical Pharmacy, Heidelberg University, Heidelberg, Germany; ^4^ Department of Cardiac Surgery, Heidelberg University Hospital, Heidelberg, Germany; ^5^ Department of Cardiology, University Hospital Heidelberg, Heidelberg, Germany

**Keywords:** *Staphylococcus aureus*, comparative genomic, phage, bloodstream infection, device related infection, immune evasion cluster, *scn*, *sak*

## Abstract

Persistent infections caused by *Staphylococcus aureus* remain a clinical challenge. Adaptational mechanisms of the pathogen influencing infection persistence, treatment success, and clinical outcome in these types of infections by *S. aureus* have not been fully elucidated so far. We applied a whole-genome sequencing approach on fifteen isolates retrieved from a persistent *S. aureus* infection to determine their genetic relatedness, virulome, and resistome. The analysis of the genomic data indicates that all isolates shared a common clonal origin but displayed a heterogenous composition of virulence factors and antimicrobial resistance. This heterogeneity was reflected by different mutations in the *rpoB* gene that were related to the phenotypic antimicrobial resistance towards rifampicin and different minimal inhibitory concentrations of oxacillin. In addition, one group of isolates had acquired the genes encoding for staphylokinase (*sak*) and staphylococcal complement inhibitor (*scn*), leading to the truncation of the hemolysin b (*hlb*) gene. These features are characteristic for temperate phages of *S. aureus* that carry genes of the immune evasion cluster and confer triple conversion by integration into the *hlb* gene. Modulation of immune evasion mechanisms was demonstrated by significant differences in biofilm formation capacity, while invasion and intracellular survival in neutrophils were not uniformly altered by the presence of the immune evasion cluster. Virulence factors carried by temperate phages of *S. aureus* may contribute to the course of infection at different stages and affect immune evasion and pathogen persistence. In conclusion, the application of comparative genomic demonstrated clonal heterogeneity in persistent *S. aureus* infection.

## Introduction

*Staphylococcus aureus* is one of the most prevalent and clinically relevant pathogens and exerts a wide repertoire of virulence factors (Tong et al., 2015). Bloodstream infection (BSI) is one of the major manifestations and may, among other foci, be caused by infected foreign material. *S. aureus* infections associated with foreign bodies like prosthetic joints, heart valves, and implanted devices often result in recurrent or persistent infections, thus posing a challenge to clinicians (Thwaites et al., 2011).

The ability of *S. aureus* to cause recurrent or persistent infections has been attributed to several pathogenic mechanisms. Biofilm formation is a key component of *S. aureus* virulence in infections involving foreign bodies by establishing a protected niche where persistence even during antibiotic therapy may be facilitated (Moormeier and Bayles, 2017). Besides biofilm formation, intracellular persistence *via* the formation of small colony variants (SCV) is often associated with device-associated infections (Proctor et al., 2006; Kahl et al., 2016; Tuchscherr et al., 2020). It has been proposed that intracellular survival in leukocytes can lead to dissemination *via* the bloodstream (Thwaites and Gant, 2011; Mulcahy et al., 2020; Pidwill et al., 2020).

One of the hallmarks of *S. aureus* as a successful pathogen is the presence of virulence factors on mobile genetic elements, such as bacteriophages and genomic islands, which contribute to the diversity of *S. aureus*. *S. aureus* bacteriophages are widely distributed and known to carry virulence factors like Panton-Valentine leukocidin, staphylococcal enterotoxin A, staphylokinase, staphylococcal complement inhibitor, or chemotaxis inhibitory protein (van Wamel et al., 2006; Goerke et al., 2009; Ingmer et al., 2019). While it has been demonstrated that blood culture isolates lacking staphylokinase were significantly associated with worse clinical outcome (Jin et al., 2003), few studies have investigated the involvement of bacteriophages during the course of infection, where bacteriophages carrying virulence factors may have influenced infection persistence, progress, and outcome (Goerke et al., 2004; Boyle-Vavra et al., 2011).

In the present study, we analyzed fifteen clinical *S. aureus* isolates from a patient with multiple episodes of BSI and an infected implantable cardioverter-defibrillator (ICD) over a period of eleven months by whole genome sequencing (WGS) to investigate the genetic relatedness, virulome, and resistome to gain insight into evolutionary mechanisms in this type of infections.

## Methods

### Diagnostic Microbiology and Patient Data

Blood cultures and swabs were processed during routine patient care and diagnostic microbiology was performed according to current standards. Briefly, MALDI-TOF (Bruker, Germany) was used for species identification, Vitek2 (bioMérieux, Germany) or minimal inhibitory concentration (MIC) test strips (Liofilchem, Italy) for antibiotic susceptibility testing (AST) and interpreted to EUCAST guidelines in the version valid at the time of isolation. Bacterial isolates were cryopreserved until further analyses. Patient characteristics and data were extracted from the medical record and anonymized for publication.

### DNA Extraction and Whole Genome Sequencing (WGS)

Genomic DNA was extracted from overnight bacterial culture on Columbia blood sheep agar (BD, Germany) using the DNeasy Blood and Tissue Minikit (Qiagen GmbH, Germany) according to manufacturer’s protocol with the addition of prior lysis step with lysostaphin (final concentration 25 µg/ml, Genaxxon GmbH, Germany). The library was prepared using Illumina NextFlex kit and sequenced with the Illumina MiSeq (2x300). Quality control, assembly, annotation, and typing were performed as described previously (Kocer et al., 2019).

Briefly, raw sequences were trimmed for quality using Sickle 1.33 (parameters, q>30; 1>45) (Joshi and Fass, 2011). The cleaned sequences were then assembled using SPAdes 3.13.0 (Bankevich et al., 2012). Assemblies were curated for length (>1000 bp) and coverage (>10×) to ensure no errors and contamination in the draft genome. Coverage of the genome was evaluated using Samtools (Li et al., 2009). Annotation was performed using Prokka 1.14.1 (based on Genetic Code Table 11) (Seemann, 2014). Core genome analysis was performed using Roary 3.12 (Page et al., 2015) (the core genome is defined by genes present in all the isolates) and SNPs were curated using Gubbins (Croucher et al., 2015). Virulence genes were found using an in-house database specialized in the virulence factor of *S. aureus* (genes details are provided in the **Supplementary Material**), the identification was performed using blastn and the following parameter: identity threshold of 90% and minimum coverage of 80% and prophages were detected using Phaster (Arndt et al., 2016). MLST was performed using the pubMLST database (Jolley and Maiden, 2010). A list of all SNPs within the core genome, the gene presence and absence in the accessory genome, sequence statistics and accession numbers are provided in the **Supplementary Material** and **Supplementary Tables 1–3**.

### DNA Extraction and RT-qPCR

DNA for real-time quantitative PCR (RT-qPCR) was extracted from overnight cultures on Columbia blood agar out of the cryostock. Bacteria were suspended in TE-Buffer pH 8.0 with lysostaphin (final concentration 25 µg/ml) and incubated for 30 min at 37°C until the suspension was clear. Then, proteinase K (100 µg/ml final concentration) in TE-Buffer was added and incubated for 15-20 min at 37°C. Then, the mixture was heated for 5 min at 95°C. RT-qPCR of the circular form (cf) of the bacteriophage, the phage-free chromosomal integration site (*attB*), and *dnaA* as control were performed with BiolineSensiFast SybrGreen Rox Mix according to the manufacturer’s instructions. Primer sequences were derived from (Guerillot et al., 2019).

### Growth Curve

Growth measurements were performed as triplicates on a 96-well microplate, with each well containing 150 µl of bacterial suspension in tryptic soy broth (TSB; BD) with a final inoculum of 5 x 10^5^ CFU/ml. The plate was incubated with shaking every five minutes for 15 hours at 37°C in a FLUOstar Optima (BMG Labtech) plate reader and optical density at 590 nm (OD_590_) was measured every five minutes.

### Colony Morphology and Testing for Auxothrophy

Bacterial isolates were tested for auxotrophy in the presence of menadione, thymidine, hemin, and CO_2_ as described elsewhere (Maduka-Ezeh et al., 2012). Briefly, testing for auxotrophy was done on Mueller-Hinton agar II (biomérieux, Germany) with impregnated discs containing either hemin (Sigma-Aldrich), menadione (25 µg/ml, 20 µl per disc; Sigma Aldrich) or thymidine (100 µg/ml, 20 µl per disc; Sigma Aldrich). To address whether CO_2_ has an influence on micromorphology of the isolates, they were incubated with or without 5% CO_2_ on Columbia blood sheep agar at 37°C overnight and appearance of the colonies was compared visually.

### Invasion and Intracellular Survival Assay

Invasion and intracellular survival were investigated using differentiated HL-60 neutrophils. Cells were cultured in Roswell Park Memorial Institute (RPMI) medium (anprotec, Germany) supplemented with 10% fetal calf serum (anprotec) and 1% penicillin/streptomycin (Sigma Aldrich, Germany) and differentiated by addition of 190 µl dimethyl sulfoxide (DMSO, Serva, Germany) to 10^6^ cells in 15 ml RPMI following incubation for 5 days in 5% CO_2_ containing atmosphere (Hauert et al., 2002). Differentiated HL-60 were then infected with bacterial suspensions in RPMI without penicillin/streptomycin from overnight cultures at MOI of 100 and a cell density of 7.5x10^4^ for two hours. Then, gentamicin was added at a concentration of 100 µg/ml to kill extracellular bacteria and incubated for 1 h to allow the intracellular killing of the bacteria by the neutrophils. Cells were then washed twice with PBS and lysed with 0.1% Triton-X 100. Intracellular bacteria were counted using the drop plate method, as described earlier (Herigstad et al., 2001). In brief, the cell lysates were diluted 1:100, 1:200, 1:500 and 1:1000 in phosphate buffered saline (PBS, anprotec). Two Columbia agar plates per lysate were divided into four quadrants and 10 µl of the prepared dilutions were pipetted onto the plate as drops. Per dilution, five drops were pipetted on one quadrant of one plate. The same procedure was repeated on the second plate. Following overnight incubation at 37°C, the dilution giving three to 30 colonies per drop was used for colony counting, expressed in CFU/ml.

### Biofilm Production Assay

Bacteria were cultured in TSB with 1% glucose overnight at 200 rpm. Then, bacterial cultures were adjusted to 0.5 McFarland standard in 0.9% NaCl and diluted 1:50 in TSB with 1% glucose. 100 µl bacterial suspension was then incubated in a 96-well plate overnight. After incubation, the supernatant was discarded and the plate was washed with Milli-Q water (Merck, Germany) twice. 125 µl 0.1% crystal violet was added to the wells and incubated for 10 min at room temperature. The supernatant was discarded and the plate was again rinsed with Milli-Q water twice. After drying overnight, 150 µl acetic acid was added to the wells, incubated for 10 min at room temperature, and then transferred to another 96-well plate for OD measurement at 550 nm. Experiments were performed as triplicates.

### Testing for β-Haemolysin Production

Isolate B3 was cultured from the cryostock on sheep blood agar containing 5% sheep blood (BD). Subcultures of single colonies were prepared on sheep blood agar cultured at 37°C overnight. Then, cultures were incubated at 4°C for at least 16 hours. Comparison of the hemolysis around the colonies before and after incubation at 4°C allows for the detection of β-hemolysin production (hot-cold effect) (Kmieciak et al., 2016). For the reverse CAMP test, the analyzed colonies were streaked perpendicularly to *Streptococcus agalactiae* ATCC 27956 and incubated at 37°C overnight. *S. aureus* ATCC 25923 was used as positive control and isolates B1 and B2 for comparison. An enlarged hemolysis zone, called “arrowhead”, near the *S. agalactiae* was considered positive.

### Data Presentation and Statistics

GraphPad Prism 5.0 (San Diego, CA, USA) and R 4.1.0 were used for data presentation and statistical analyses.

### Ethical Considerations

The local ethical committee was consulted for the use of bacterial isolates from patient specimen, who waived individual informed consent for the use of the bacterial isolates without patient data (S-187/2017). The patient gave written informed consent on the publication of anonymized patient data.

## Results

### *S. aureus* Isolates, Genetic Background and Clonality

Fifteen *S. aureus* clinical isolates from a 74-year-old, male patient with multiple episodes of *S. aureus* BSI over a period of 11 months were analyzed in this study. A detailed description of the clinical course is provided in the **Supplementary Material** and **Supplementary Figure 1**.

**Figure 1:**
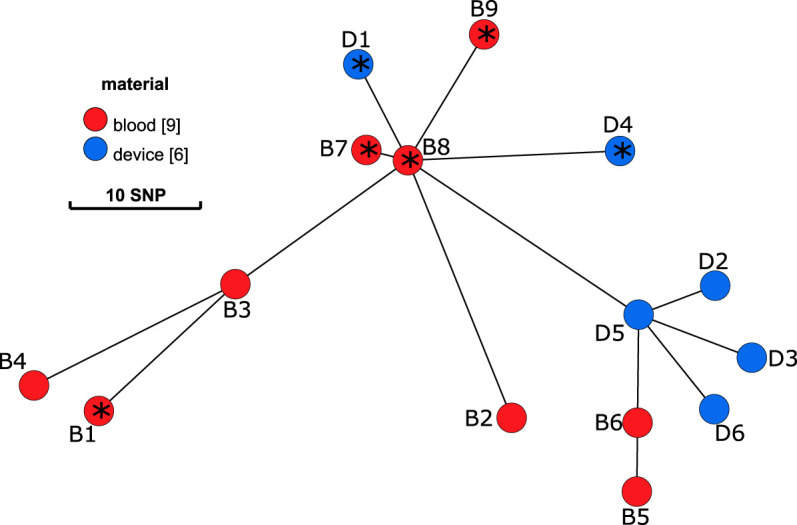
Minimum spanning tree on the sequences of *S. aureus* isolates. Blood culture isolates (B1-B9) are displayed in red and isolates from the internal cardioverter defibrillator (device, D1-6) in blue. Distance between nodes in SNPs. According to results of the WGS, isolates with an * present an insertion of a prophage within the gene *hlb*.

Isolates from blood culture (B1-B9) and from the cardiac device (D1-D6) were subjected to WGS to determine their clonal relationship, virulome, and resistome. All isolates belonged to ST8 (*spa*-type t008) and were genetically closely related with 1 to 33 SNPs within 2445 genes (2243970 nucleotides). The minimum spanning tree (MST) demonstrates a diversification from a clonal origin of all isolates (**Figure 1**). Isolates from the device are in close relationship to early blood culture isolates that were isolated during a later stage of infection. D1 and D4 are in proximity of B7-B9 and D2, D3, D5 and D6 are closer related to B5 and B6.

### Virulome and Detection of a Prophage

**Figure 2:**
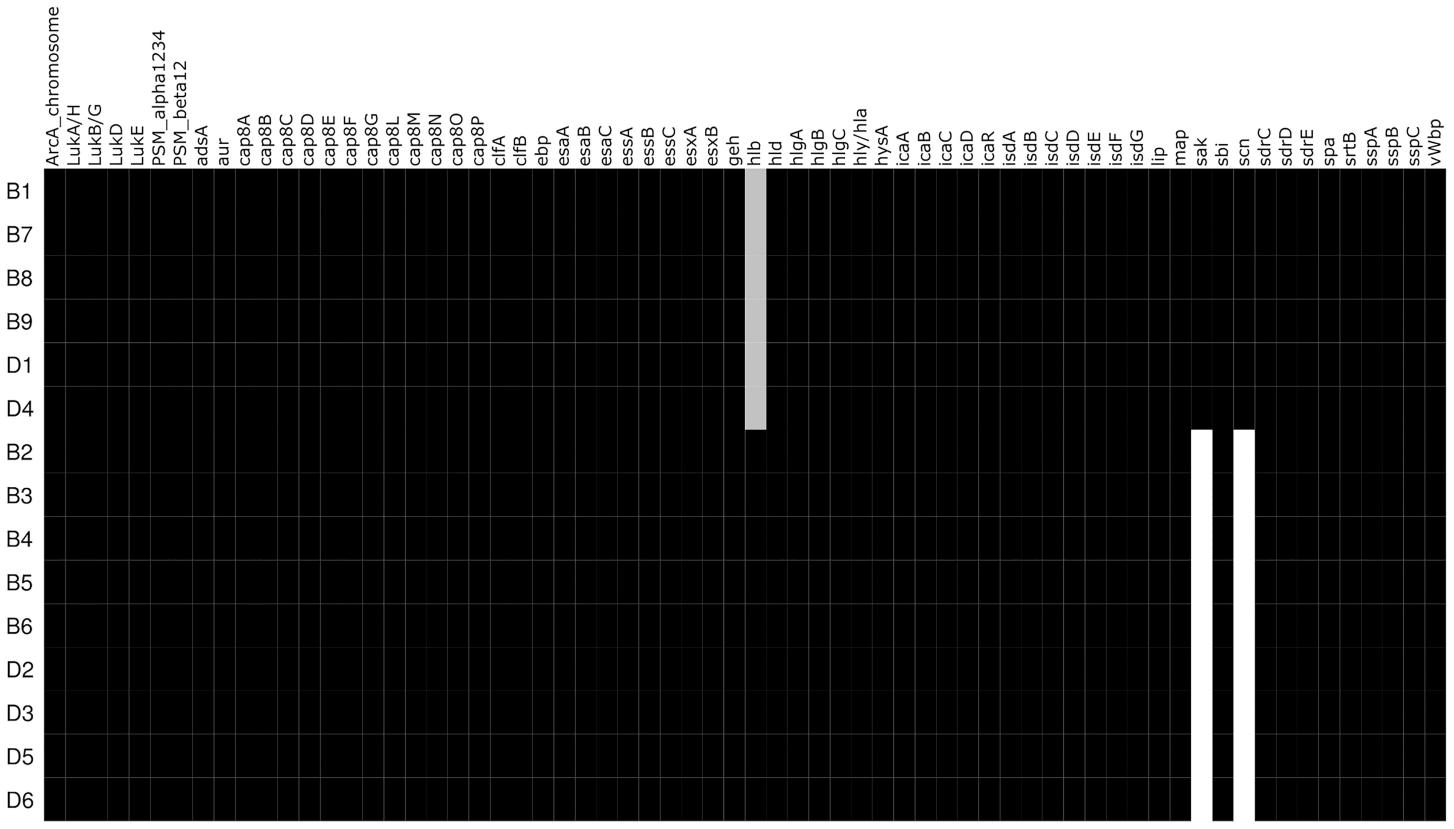
Heatmap of detected virulence genes. Virulence factors that are present in the respective isolates are black while white represents absence. Grey shading represents a truncated gene.

**Figure 3:**
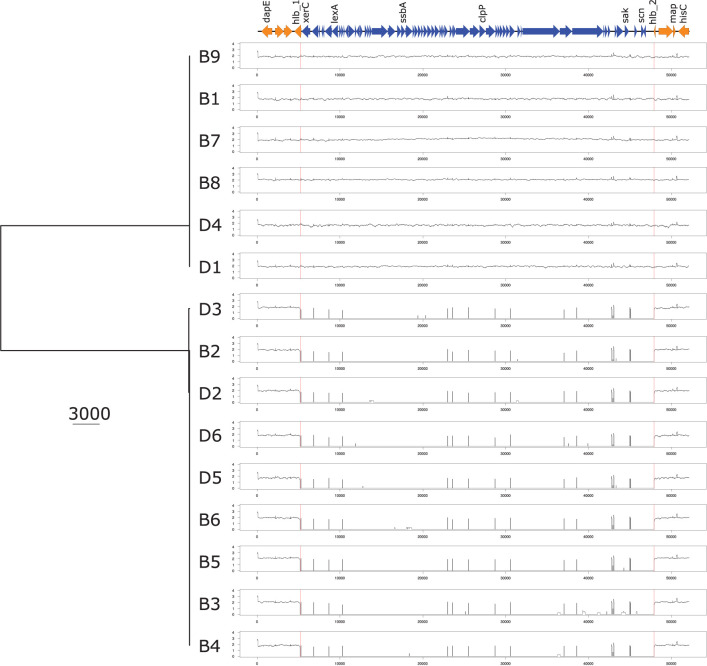
UPGMA phylogenetic tree based on the nucleotide sequence of the prophage and the surrounding genomic context. UPGMA phylogenetic tree based on the nucleotide sequence of the prophage and the *hlb* genetic environment containing the integration site of the prophage based on reads mapping analysis. *dapE*, probable succinyl-diaminopimelate desuccinylase; *hlb*, hemolysin b; *xerC*, tyrosine recombinase; *lexA*, LexA repressor; *ssbA Staphylococcus aureus*, single-stranded DNA-binding protein; *clpP*, ATP-dependent Clp protease proteolytic subunit; *sak*, staphylokinase; *scn*, staphylococcal complement inhibitor; *map*, Major histocompatibility complex class II analogue protein; *lucC*, siderophore biosynthesis protein.

Analyses of the virulome identified two groups of isolates with distinct virulence profiles (**Figure 2**). One group (B1, B7, B8, B9, D1, D4) harbored the genes encoding staphylokinase (*sak*) and staphylococcal complement inhibitor (*scn*) while the gene encoding β-hemolysin (*hlb*) was truncated. *sak* and *scn* are known to be part of the immune evasion cluster (IEC) that can be found in the group of Sa3int bacteriophages that integrate into the *hlb* gene, thus leading to truncation of *hlb* (Coleman et al., 1989; van Wamel et al., 2006). The other group (B2, B3, B4, B5, B6, D2, D3, D5, D6) did not possess *sak* and *scn*, but an intact *hlb* gene. IEC^+^ -isolates also contained *xerC*, *lexA*, *ssb*, and *clpP* along with *sak* and *scn*, and *hlb* is divided (termed *hlb_1* and *hlb_2*) by the insertion of these genes into the chromosome, as displayed in **Figure 3** and **Supplementary Figure 2**. The identical coverage between the phage part and the chromosomal context upstream and downstream of the *hlb* gene associated with the complete absence of the phage genes in the IEC^-^ isolates suggested that the insertion only occurred within that genomic context. The genetic context of the prophage is displayed in the **Supplementary Material** and **Supplementary Figure 2**. Phaster analysis only detected one intact prophage and one questionable prophage, which could also be another mobile genetic element. This second MGE most likely is a *Staphylococcus aureus* pathogenicity island (SaPI); it is identical in all isolates and located in the same genomic context (**Supplementary Material** and **Supplementary Figure 3**). The integrase of the intact prophage was 99% identical to the integrase of the prophage ɸ13 belonging to the group of Sa3int phages, and is a tyrosine recombinase-type.

**Figure 4:**
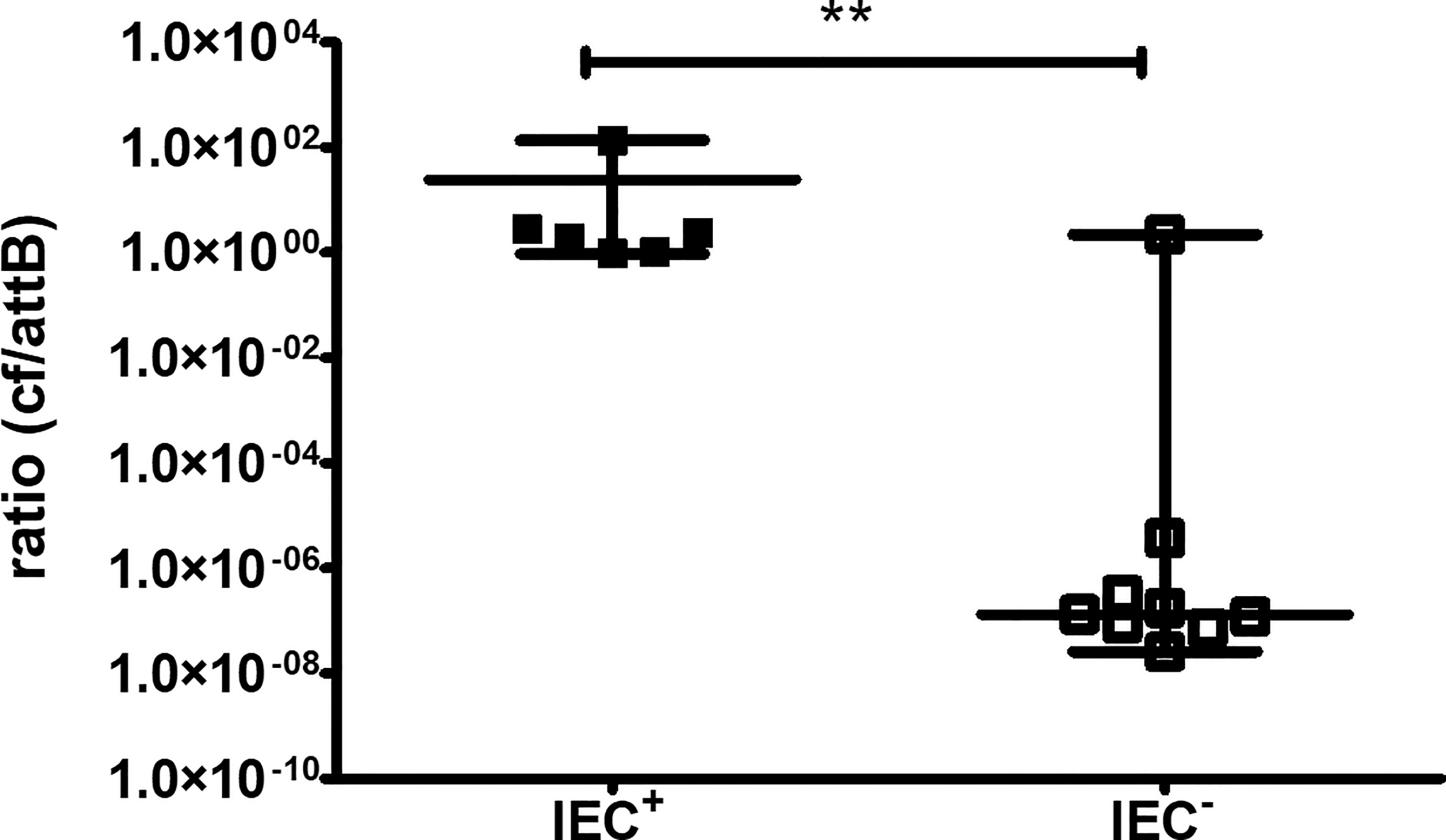
RT-qPCR for the circular form of the bacteriophage and the phage-free chromosomal integration site (*attB*). The circular form (cf) of the bacteriophage and phage-free chromosomal site (*attB*) were detected by PCR. Isolates are regarded as IEC^+^ and IEC^-^ according to WGS data. Results of three independent experiments performed as duplicates. The comparison of two data groups were analyzed by Mann–Whitney U test (one-tailed, confidence intervals 95%) with **P < 0.01.

As WGS data demonstrated differences in the genomic content that pointed to the presence or absence of a bacteriophage in the two groups, we aimed to confirm the presence using RT-qPCR. The circular form of the phage, meaning it is integrated into the genome as a prophage or is a free circular form, and the phage-free chromosomal integration site (*attB*) corresponding to the absence of the phage from the genome can be detected by different PCRs, as published by Guerillot et al. (Guerillot et al., 2019). The ratio of the circular phage to the *attB* site was significantly higher in IEC^+^-isolates than in IEC^–^isolates (P ≤ 0.01, **Figure 4**). However, there was one outlier in the IEC^-^ isolates, B3.

WGS results showed that B3 was negative for IEC-associated genes but PCR detected high levels of a circular form of the phage. Screening of the raw fastq files for integration of the prophage at a different site was negative.

### Resistome and Antimicrobial Resistance

According to the virulome, there were two groups with distinct virulence gene profiles. However, we did not observe the identical two groups when analyzing the antimicrobial resistance (AMR) (**Table 1**). One isolate (B2) displayed a wild-type *rpoB* gene, while the majority of isolates (n=11/15, 73%) harbored an Asp471Glu mutation, two isolates (B3, B4) a Ser464Pro mutation and isolate B1 His 481Tyr. B1, B3, and B4 were also phenotypically resistant towards rifampicin (MIC ≥32 mg/L, 16 mg/L and 6 mg/L, respectively).

**Table 1:** Phenotypic antimicrobial resistance and detected resistance genes.

Isolate	phenotypic AMR	AMR genes	*rpoB* mutation
B1	rifampicin	*(AGly)apH, norA, lmrS, fosB, mepA, mepR, tet(38)*	His481Tyr
B2	fosfomycin	*(AGly)apH, norA, lmrS, fosB, mepA, mepR, tet(38)*	none
B3	rifampicin, fosfomycin	*(AGly)apH, norA, lmrS, fosB, mepA, mepR, tet(38)*	Ser464Pro
B4	rifampicin, fosfomycin	*(AGly)apH, norA, lmrS, fosB, mepA, mepR, tet(38)*	Ser464Pro
B5	fosfomycin	*(AGly)apH, norA, lmrS, fosB, mepA, mepR, tet(38)*	Asp471Glu
B6	fosfomycin	*(AGly)apH, norA, lmrS, fosB, mepA, mepR, tet(38)*	Asp471Glu
B7	fosfomycin	*(AGly)apH, norA, lmrS, fosB, mepA, mepR, tet(38)*	Asp471Glu
B8	fosfomycin	*(AGly)apH, norA, lmrS, fosB, mepA, mepR, tet(38)*	Asp471Glu
B9	fosfomycin	*(AGly)apH, norA, lmrS, fosB, mepA, mepR, tet(38)*	Asp471Glu
D1	fosfomycin	*(AGly)apH, norA, lmrS, fosB, mepA, mepR, tet(38)*	Asp471Glu
D2	fosfomycin	*(AGly)apH, norA, lmrS, fosB, mepA, mepR, tet(38)*	Asp471Glu
D3	oxacillin, fosfomycin	*(AGly)apH, norA, lmrS, fosB, mepA, mepR, tet(38)*	Asp471Glu
D4	fosfomycin	*(AGly)apH, norA, lmrS, fosB, mepA, mepR, tet(38)*	Asp471Glu
D5	oxacillin, fosfomycin	*(AGly)apH, norA, lmrS, fosB, mepA, mepR, tet(38)*	Asp471Glu
D6	fosfomycin	*(AGly)apH, norA, lmrS, fosB, mepA, mepR, tet(38)*	Asp471Glu

(AGly)apH aminoglycoside resistance gene; norA multidrug resistance efflux pump gene norA; lmrS multidrug efflux pump lmrS; fosB; mepA and mepR multidrug efflux pump gene; tet(38) tetracyclin resistance gene; rpoB RNA polymerase beta subunit.

While there was no additional difference in the detected resistance genes (**Table 1**), some differences in phenotypic AMR among the isolates were observed. Neglecting minor deviations in MIC values that may be explained by technical variations and are not leading to differences in interpretation, we detected heterogenous susceptibility towards oxacillin, fosfomycin, and rifampicin, as mentioned above. Resistance towards fosfomycin was present in 14 of 15 isolates (MIC ≥128 mg/L) with only isolate B1 being susceptible (MIC ≤8 mg/L), while *fosB* was present in all isolates. Isolates D3 and D5 had a MIC for oxacillin of 16 mg/L and 12 mg/L, respectively. Neither *mecA* nor *mecC* was detected in any of the isolates. The complete results of phenotypic AST can be found in the **Supplementary Material** and **Supplementary Table 4**.

### Growth Characteristics and Testing for Auxothrophy

We observed differences in colony diameter, pigmentation, and hemolysis among the isolates (**Supplementary Material** and **Supplementary Figure 2**). However, this morphology did not correlate with the presence or absence of genes of the IEC. To test whether the presence of genes of the IEC is associated with small colony variant (SCV), the isolates were analyzed for growth and auxotrophy, which is a key feature of SCV (Kahl et al., 2016; Tuchscherr et al., 2020). Although the isolates showed different growth characteristics, there was no significant difference between the two groups (**Supplementary Material** and **Supplementary Figure 5**). Looking at the growth curves, we observed that there was a group of slower growing isolates and faster growing isolates independent of the presence of the IEC. The “slow growers” phenotype is associated with a cluster of isolates in the minimum spanning tree constructed from the core genome (**Figure 1**, isolates B5, B6, D2, D3, D5 and D6). We identified non-synonymous modifications within genes encoding for the proteins 50S ribosomal protein L6, response regulator protein GraR, teichoic acid D-alanine hydrolase, cell division protein DivIB, GTPase Der and NAD-dependent malic enzyme in isolates B5, B6, D2, D3, D4 and D6 while the isolates B1, B2, B3, B4, B7, B8, B9, D1, D4 are unaltered. Especially the alteration of the gene DivIB, which is involved in bacterial cytokinesis (Wadsworth et al., 2008), may be relevant in the context of differences in growth characteristics in the respective isolates. Isolates were tested for auxotrophy in the presence of menadione, thymidine, hemin, and CO_2_. No alterations in the growth characteristics could be observed in the presence of any of the tested substances or CO_2_ (**Supplementary Material**, **Supplementary Figure 6**).

### Invasion Into Neutrophils, Intracellular Survival and Biofilm Production

**Figure 5:**
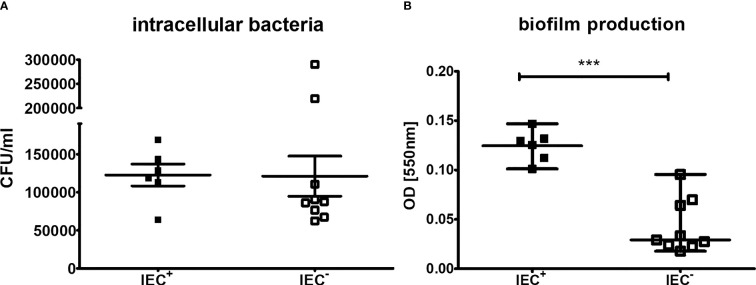
Invasion and intracellular survival in differentiated neutrophils and biofilm production of clinical isolates. Capacity of *S. aureus* clinical isolates to **(A)** invade neutrophils and survive intracellular and **(B)** form biofilm. **(A)** For measurements of invasion and intracellular survival, differentiated HL-60 were incubated with *S. aureus* at a MOI of 100 2h at 37°C. Extracellular bacteria were killed using gentamicin and cells were lysed to detect intracellular bacteria following incubation for 1h to allow intracellular killing. The outliers are isolates B3 and D6. Results of three independent experiments. **(B)** Biofilm formation was measured using a crystal violet stain, experiments performed as triplicates. The comparison of two data groups were analyzed by Mann– Whitney U test (one-tailed, confidence intervals 95%) with ***P < 0.001.

As it has been demonstrated that the presence of the IEC can lead to enhanced biofilm production and invasion (Rooijakkers et al., 2006; Sultan et al., 2018; Guerillot et al., 2019), we tested the invasiveness into neutrophils and the intracellular survival using differentiated HL-60 neutrophils. After incubation of the neutrophils with the bacterial isolates and killing of extracellular bacteria with gentamicin, intracellular bacteria were counted using the plate drop method following cell lysis. However, comparing the IEC^+^- and IEC^–^groups, we did not detect a significant difference (**Figure 5A**, P=0.16). There are two outliers in the IEC^-^ group, isolates B3 and D6. Interestingly, B3 also showed high levels of a circular form of the phage in RT-qPCR.

To determine the capacity to form a biofilm of the *S. aureus* isolates, a biofilm assay with crystal violet staining was performed. IEC^+^-isolates produced significantly more biofilm compared to IEC^–^isolates (**Figure 5B**, P<0.001).

### Testing for β-Hemolysis

As isolate B3 showed differences in the results of WGS and qPCR, testing for β-hemolysis of single colonies from isolate B3 was performed and revealed that there were β-hemolysin positive and negative subpopulations, as determined by hot-cold effect and reverse CAMP test. Subculturing of single colonies from these subpopulations showed that colonies without β-hemolysis were able to convert to β-hemolysin production spontaneously. This points to a relative plasticity of a subpopulation of this isolate, as shown in two examples in the **Supplementary Material**, **Supplementary Figure 7**.

## Discussion

WGS of all available clinical *S. aureus* isolates demonstrated a common clonal origin. Albeit clonality, differences in the virulome and resistome were observed. Two groups of isolates were identified by the presence or absence of the virulence genes *sak* and *scn*, and the truncation of *hlb*. These characteristics indicate the involvement of *S. aureus* temperate bacteriophages carrying genes of the IEC as they insert into the β-hemolysin gene and thereby confer conversion by disrupting *hlb* and inserting other virulence factors (Coleman et al., 1989; van Wamel et al., 2006). We detected *sak* and *scn*, but not *chp* or other virulence genes in the clinical isolates. Although the occurrence of *sak* in Sa7int phages has been demonstrated (Goerke et al., 2009; Ingmer et al., 2019), the findings are a typical feature of Sa3int phages and we detected an integrase 99% identical to the integrase of the prophage ɸ13, belonging to the Sa3int group, which is a tyrosine recombinase type. Thus, we conclude that the phage belongs to the Sa3int group.

It has been demonstrated that β-hemolysin plays a role in regulating inflammation, thereby enabling vegetation formation and disease progression in infective endocarditis (Herrera et al., 2017). The insertion of the phage leads to loss of the function of β-hemolysin (Coleman et al., 1989; Tran et al., 2019); At the same time, integration leads to the expression of *scn* and *sak*, that are located on the prophage and may be beneficial due to a higher capacity to form a biofilm, invade cells and evade host immunity (Rooijakkers et al., 2006; Sultan et al., 2018). Indeed, we were able to demonstrate a higher capacity of the IEC^+^-isolates to form biofilm. Biofilm formation is a key element of infections involving foreign bodies (Rooijakkers et al., 2006; Tong et al., 2015; Moormeier and Bayles, 2017), and expression of the IEC may support the establishment of the infection in this situation. On the other hand, it has been demonstrated that biofilm formation can induce phage excision *in vitro* (Tran et al., 2019). IEC^+^-isolates were detected at the beginning (B1) as well as at disease progression (B7-B9) and were isolated from the ICD (D1 and D4). There was a mixed population of IEC^+^ and IEC^-^ cultured from the device pointing to a probable dynamic in the full population. In addition, we observed a composition of β-hemolysis positive and negative subpopulations in isolate B3. A mixed population for instance in a biofilm could potentially be beneficial for the bacterial population as it would permit the expression of the virulence factors encoded in the IEC along with β-hemolysin. That may contribute to the successful establishment and spread of the infection.

As we observed an alternation of IEC^+^ and IEC^-^ *S. aureus* isolates in the blood cultures during the course of infection and both IEC^+^ and IEC^-^ isolates were cultured from the device, and we detected β-hemolysin positive and negative subpopulations in B3, it is reasonable to consider a mixed bacterial population in a single infective focus. There are different ways diagnostic procedures may lead to the detection of clonally related isolates with differences in virulence genes in follow-up cultures. First, during the procedure of taking blood, 16-20 ml per blood culture set is inoculated into the bottles. In theory, it would be sufficient if a single bacterium is inoculated into the bottle to detect growth. In this case, a mixed population of bacteria would be missed by the procedure of taking blood. Second, if a mixed population is inoculated into the blood culture bottle, the diagnostic procedures in the microbiology laboratory do not, in general, consider a mixed population. In most cases, if the macromorphology is homogeneous, one or two representative colonies would be selected for identification and antimicrobial susceptibility testing. Thus, heterogeneity would be missed in this step. Third, for cryopreservation, also one or two colonies would be selected, missing mixed bacterial populations in this step. Taken together, we cannot rule out that diagnostic and preservation procedures may have led to selection bias of clonally related isolates harboring different virulence genes over time.

The presence of IEC in *S. aureus* was linked to a SCV phenotype in a recent study (Guerillot et al., 2019). SCV may be caused by various alterations in the metabolism (Proctor et al., 2006; Kahl et al., 2016). Although we observed different phenotypes regarding colony size, pigmentation, and hemolysis in the isolates, it was not associated with the presence of the IEC nor did we detect any association of growth characteristics and testing for auxotrophy did not confirm SCV in any of the isolates.

Isolate B3 exhibited contradictory results in the RT-qPCR and WGS. Therefore, testing for β-hemolysis on multiple distinct colonies was performed using hot-cold hemolysis and CAMP assay to investigate the presence of a heterogeneous population. Indeed, both assays confirmed the presence of β-hemolysin positive and negative subpopulations, which provides a plausible explanation for the discrepancy between WGS and RT-qPCR. Of note, B3 was an outlier in the neutrophil invasion assay. Interestingly, prophage integration in a subpopulation of isolate B3 may not be stable as implied by the restoration of β-hemolysis activity through a subculturing process, supporting the idea of phenotypic plasticity through phage mobilization (Goerke et al., 2004; Goerke et al., 2006b). Besides isolate B3, isolate D6 was also an outlier in the neutrophil invasion assay. In contrast to B3, the WGS and RT-qPCR results for D6 were concordant.

*S. aureus* prophages harboring genes of the IEC are highly prevalent with 80% positive strains in a global database of over seven thousand *S. aureus* genomes (Guerillot et al., 2019). In chronic *S. aureus* infections of cystic fibrosis patients, phage mobilization leading to genome alteration and variable expression of virulence determinants have been observed in longitudinal analysis (Goerke et al., 2004; Goerke et al., 2006b). The occurrence of phage excision containing the genes of the IEC *in vivo* was also detected in one isolate from a study comparing isogenic MRSA blood culture isolates linked to daptomycin therapy failure (Boyle-Vavra et al., 2011). The authors also detected *sak* and *scn* along with *clpP* in one isolate, which is congruent with our findings as we detected the same genes in one group of the isolates.

Prophage induction and mobility are important for the evolution and virulence of *S. aureus* (Verkaik et al., 2011; Feiner et al., 2015). Prophages can be induced and are then excised from the bacterial genome by environmental conditions like DNA damage or oxidative stress (Goerke et al., 2006a; Tran et al., 2019). Exposure to antimicrobial substances, such as trimethoprim and ciprofloxacin, can lead to phage induction in *S. aureus* isolates from cystic fibrosis patients (Goerke et al., 2006a). Phage induction has been demonstrated after incubation with ciprofloxacin for only 6h (Goerke et al., 2006a). Besides stress-related response, phage dynamics may be dependent on the phage-type and *in vivo* host-pathogen interaction may be relevant. The influence of antimicrobial substances on phage induction warrants further research as the consequences that should be drawn for clinical management are unclear.

As mentioned above, we observed clonal heterogeneity in the isolates of this study. This was not only reflected by the differences in the virulome, but also in the phenotypic AMR and the presence of different mutations in the *rpoB*. These differences may be of clinical importance, as for oxacillin, we observed two isolates with oxacillin MICs that would in the absence of the *mecA* or *mecC* gene be an indication for borderline oxacillin resistant *S. aureus* (BORSA) (Hryniewicz and Garbacz, 2017). The group of anti-staphylococcal β-lactam antibiotics including isoxazolyl penicillins are the first-line treatment for methicillin-susceptible *S. aureus* infections in patients without penicillin allergy (Tong et al., 2015). However, the presence of BORSA may increase the risk for treatment failure (Hryniewicz and Garbacz, 2017), especially if this is not detected during the course of a persistent and/or recurrent infection. Likewise, rifampicin showed heterogenous results in the AST and for rifampicin different mutations in the *rpoB* were observed. Mutations leading to amino acid substitutions of His481Tyr and Ser464Pro have been linked to phenotypic resistance of rifampicin earlier (Aubry-Damon et al., 1998; Wichelhaus et al., 1999), and we detected this in our phenotypic resistant isolates. Amino acid substitution Asp471Glu did not result in resistance towards rifampicin. This mutation was described in rifampicin-resistant *S. epidermidis* isolates, but only in combination with a different mutation in *rpoB* (Albano et al., 2019). For fosfomycin, we did not find a genetic correlate for the phenotypic resistance in 14 of 15 isolates with one isolate being susceptible. Rifampicin and fosfomycin are known to exert an anti-biofilm effect and are often used in combination with other antibiotic substances to treat infection with foreign material involved (Mihailescu et al., 2014; Tong et al., 2015). Thus, heterogeneity in antimicrobial susceptibility towards these substances could possibly impact clinical outcomes and treatment success. In conclusion, microbial diagnostics and AST need to be performed carefully in these cases, as heterogeneity in the bacterial population should not be overlooked.

As a limitation, the *S. aureus* isolate of the first BSI was not available for analysis. Thus, we are not able to investigate the evolution and clonal relationship between the initial infection isolate and subsequent isolates. In addition, as the study was conducted on cryopreserved clinical isolates from the BSI, we cannot rule out that genomic heterogeneity in these samples may have been missed by diagnostic procedures. Nevertheless, the longitudinal nature of the case allowed us to gain insight into the dynamics, evolution, and heterogeneity of bacterial population during persistent infection. This is also a strength of the study, as we provide analyses on an 11-month course of infection with 15 clonally related but heterogenous clinical isolates.

In conclusion, this study demonstrates by the use of genomic comparison of *S. aureus* isolates a clonal heterogeneity in a persistent *S. aureus* infection. This heterogeneity was observed in the virulome and resistome. Involvement of prophages carrying virulence factors and with different capacities to form biofilms, as well as changes in antimicrobial susceptibility pattern, may have a clinical impact by facilitating persistent and/or recurrent infections or treatment failure. Further studies are needed that focus on heterogeneity of ICD or device related infection and longitudinal dynamics in this type of infection, as they remain a clinical challenge. The role of different antibiotic substances or other agents that may influence phage dynamics should be investigated more closely, as this may be a relevant factor for therapy success.

## Data Availability Statement

The datasets presented in this study can be found in online repositories. The names of the repository/repositories and accession number(s) can be found in the article/**Supplementary Material**.

## Ethics Statement

Written informed consent was obtained from the individual(s) for the publication of any potentially identifiable images or data included in this article.

## Author Contributions

SK, DN, SB, and KH contributed to the conception of the study. DW, PS, BM, and SK were involved in patient care and management. SK, SB, BM, and DW acquired patient-related data. SB, DN, and SK analyzed WGS data and provided visualization of the results. AS and SK performed wet lab experiments. SK, DN, and SB drafted the manuscript. All authors revised the manuscript for intellectual content and consented the final version to be submitted.

## Conflict of Interest

The authors declare that the research was conducted in the absence of any commercial or financial relationships that could be construed as a potential conflict of interest.

## Publisher’s Note

All claims expressed in this article are solely those of the authors and do not necessarily represent those of their affiliated organizations, or those of the publisher, the editors and the reviewers. Any product that may be evaluated in this article, or claim that may be made by its manufacturer, is not guaranteed or endorsed by the publisher.
