# Supplementary material for: Comparative Genomic Reveals Clonal Heterogeneity in Persistent Staphylococcus aureus Infection
Source: Front Cell Infect Microbiol. 2022 Feb 21;12:817841. doi: 10.3389/fcimb.2022.817841 (PMC8900520; doi:10.3389/fcimb.2022.817841)
Supplement: Supplementary file 1 [file DataSheet_1.pdf]

Supplementary Material

1 Supplementary methods

Virulence genes of the in-house database used in this study (listed in alphabetical order):

abi-alpha;adsA;aliD;arcA;ArcA_ACME;ArcA_chromosome;arcB;arcC;arcD;argR;aur;cap8A;cap8B;cap8C;cap8D;cap8E;cap8F;cap8G;cap8H;cap8I;cap8J;cap8K;cap8L;cap8M;cap8N;cap8;cap8P;CC_byosynthesis;chp;clfA;clfB;cna;coa;copB;copB;ebp;esaA;esaB;esaC;essA;essB;essC;esxA;esxB;eta;etb;fnbA;fnbB;geh;hlb;hld;hlgA;hlgB;hlgC;hly/hla;hysA;icaA;icaB;icaC;icaD;icaR;isdA;isdB;isdC;isdD;isdE;isdF;isdG;lip;LukA/H;LukB/G;LukD;LukE;lukF-PV;lukS-PV;LysR-like;map;mco;merA;merB;;merR;MSF-transporter;NPD-like;opp-3A;opp-3B;opp-3C;PSM_alpha1234;PSM_beta12;sak;sbi;scn;sdrC;sdrD;sdrE;sea;seb;sec;sed;see;seh;selk;sell;selq;spa;speG;srtB;sspA;sspB;sspC;TR;tsst-1;vWbp;YdhK.

2 Supplementary Tables

Supplementary Table 1: Sequencing statistics and accession numbers.

isolate	accession	coverage	# contigs	Largest contig	Total length	GC (%)	N50	N75	L50	L75
**B1**	SAMN20347825	34	18	971877	2731537	32,69	942749	179218	2	3
**B2**	SAMN20347826	48	13	1281244	2692723	32,68	993523	993523	2	2
**B3**	SAMN20347827	68	18	971878	2689483	32,68	866250	299695	2	3
**B4**	SAMN20347828	37	15	993536	2689532	32,68	872300	299677	2	3
**B5**	SAMN20347829	65	14	993519	2692905	32,68	823025	458390	2	3
**B6**	SAMN20347830	51	16	993519	2690941	32,68	823025	299938	2	3
**B7**	SAMN20347831	71	14	993524	2735005	32,69	866202	414580	2	3
**B8**	SAMN20347832	70	16	971866	2732332	32,69	866232	299673	2	3
**B9**	SAMN20347833	27	18	971846	2731813	32,69	866232	299691	2	3
**D1**	SAMN20347834	33	18	971846	2732157	32,69	866232	299691	2	3
**D2**	SAMN20347835	32	15	993514	2691264	32,68	866226	407317	2	3
**D3**	SAMN20347836	32	14	993519	2691969	32,68	866082	414579	2	3
**D4**	SAMN20347837	21	20	835848	2732605	32,69	327183	172966	3	5
**D5**	SAMN20347838	28	14	993514	2691444	32,68	980021	299938	2	3
**D6**	SAMN20347839	35	18	971830	2689035	32,68	866229	299694	2	3

Abbr.: For numeration of isolates, see Supplementary Figure 1.

Supplementary Table 2: List of SNPs.

CHR	POS	B1	B2	B3	B4	B5	B6	B7	B8	B9	D1	D2	D3	D4	D5	D6	EFFECT	GENE	PRODUCT
**NODE_1**	44621	C	C	C	C	T	T	C	C	C	C	T	T	C	T	T	missense_variant c.482G>A p.Gly161Asp	rplF	50S ribosomal protein L6
**NODE_1**	182969	C	C	C	C	C	C	T	T	T	T	C	C	T	C	C	missense_variant c.724C>T p.Pro242Ser		hypothetical protein
**NODE_1**	183464	T	T	G	G	T	T	T	T	T	T	T	T	T	T	T	missense_variant c.1219T>G p.Phe407Val		hypothetical protein
**NODE_1**	188200	C	C	C	C	C	C	C	C	T	T	C	C	C	C	C	missense_variant c.110C>T p.Thr37Ile	sarZ	HTH-type transcriptional regulator SarZ
**NODE_1**	457917	T	T	T	T	T	T	T	T	T	T	C	C	T	C	C			
**NODE_1**	458671	T	T	T	C	T	T	T	T	T	T	T	T	T	T	T	synonymous_variant c.258A>G p.Gln86Gln	arcR	HTH-type transcriptional regulator ArcR
**NODE_1**	525887	T	T	T	T	T	T	T	T	T	T	T	T	A	T	T	synonymous_variant c.60A>T p.Thr20Thr	ykoD_1	Putative HMP/thiamine import ATP-binding protein YkoD
**NODE_1**	561236	T	T	T	T	T	T	T	T	T	T	T	T	T	C	T	synonymous_variant c.660T>C p.Thr220Thr	gyrA	DNA gyrase subunit A
**NODE_1**	562432	C	C	C	C	T	T	C	C	C	C	T	T	C	T	T	missense_variant c.1856C>T p.Thr619Ile	gyrA	DNA gyrase subunit A

**NODE_1**	626418	G	G	G	G	G	G	A	A	A	A	G	G	A	G	G	stop_gained c.448C>T p.Gln150*	spa	Immunoglobulin G-binding protein A
**NODE_1**	628320	T	T	T	T	T	T	T	T	T	T	T	C	T	T	T			
**NODE_1**	752886	G	G	A	A	G	G	G	G	G	G	G	G	G	G	G	stop_gained c.684G>A p.Trp228*	uhpT	Hexose-6-phosphate:phosphate antiporter
**NODE_1**	753275	G	G	G	G	T	T	G	G	G	G	T	T	G	T	T	missense_variant c.1073G>T p.Gly358Val	uhpT	Hexose-6-phosphate:phosphate antiporter
**NODE_1**	755422	A	A	A	A	A	A	T	T	T	T	A	A	T	A	A	stop_gained c.827T>A p.Leu276*		putative sensor-like histidine kinase
**NODE_1**	887607	A	G	A	A	A	A	A	A	A	A	A	A	A	A	A	missense_variant c.478T>C p.Trp160Arg	glpT	Glycerol-3-phosphate transporter
**NODE_1**	887624	C	C	C	C	T	T	C	C	C	C	T	T	C	T	T	missense_variant c.461G>A p.Gly154Glu	glpT	Glycerol-3-phosphate transporter
**NODE_1**	960289	A	A	A	A	A	A	A	A	A	A	A	A	A	A	C	synonymous_variant c.216A>C p.Thr72Thr	ssl1	Staphylococcal superantigen-like 1
**NODE_1**	971750	T	T	T	T	T	T	T	T	T	T	T	T	C	T	T			
**NODE_11**	11467	G	G	G	G	G	G	G	G	G	G	G	G	A	G	G	missense_variant c.751G>A p.Val251Met		hypothetical protein
**NODE_11**	16959	A	A	A	A	A	A	A	A	A	A	A	A	A	A	G	synonymous_variant c.231A>G p.Ala77Ala	sepA	Multidrug resistance efflux pump SepA
**NODE_2**	79686	A	A	A	A	A	A	T	T	T	T	A	A	T	A	A			

**NODE_2**	93673	C	C	T	T	C	C	C	C	C	C	C	C	C	C	C	missense_variant c.85C>T p.His29Tyr	cspA_1	Cold shock protein CspA
**NODE_2**	93766	G	G	G	G	A	A	A	A	A	A	A	A	A	A	A	missense_variant c.178G>A p.Ala60Thr	cspA_1	Cold shock protein CspA
**NODE_2**	146090	C	C	T	T	C	C	C	C	C	C	C	C	C	C	C	missense_variant c.1411G>A p.Glu471Lys	parE	DNA topoisomerase 4 subunit B
**NODE_2**	265051	C	C	C	C	C	C	C	C	C	C	C	C	T	C	C	missense_variant c.188G>A p.Gly63Asp	topA	DNA topoisomerase 1
**NODE_2**	408124	G	A	G	G	G	G	G	G	G	G	G	G	G	G	G	stop_gained c.994C>T p.Gln332*	pycA	Pyruvate carboxylase
**NODE_2**	411085	G	C	G	G	G	G	G	G	G	G	G	G	G	G	G			
**NODE_2**	418613	G	A	G	G	G	G	G	G	G	G	G	G	G	G	G			
**NODE_2**	515535	G	G	G	G	G	G	T	T	T	T	G	G	G	G	G			
**NODE_2**	515569	C	C	C	C	C	C	T	T	T	T	C	C	T	C	C			
**NODE_2**	525857	C	T	C	C	C	C	C	C	C	C	C	C	C	C	C	missense_variant c.491C>T p.Ser164Phe		hypothetical protein
**NODE_2**	530801	T	T	T	T	T	T	C	C	C	C	T	T	C	T	T	missense_variant c.280A>G p.Ile94Val	spxA	Regulatory protein Spx
**NODE_2**	543962	A	A	G	G	A	A	A	A	A	A	A	A	A	A	A			
**NODE_2**	643649	A	T	A	A	A	A	A	A	A	A	A	A	A	A	A	stop_gained c.1182T>A p.Tyr394*	clfA	Clumping factor A

**NODE_2**	801375	G	G	G	G	A	A	G	G	G	G	A	A	G	A	A			
**NODE_2**	805464	C	C	C	C	C	C	C	C	C	C	C	C	C	C	T			
**NODE_2**	846956	T	T	T	T	T	T	T	T	T	T	T	C	T	T	T	missense_variant c.149A>G p.Lys50Arg		hypothetical protein
**NODE_2**	907981	G	G	A	A	G	G	G	G	G	G	G	G	G	G	G	missense_variant c.1739C>T p.Ala580Val	fusA	Elongation factor G
**NODE_2**	917151	A	G	G	G	G	G	G	G	G	G	G	G	G	G	G	missense_variant c.1441T>C p.Tyr481His	rpoB	DNA-directed RNA polymerase subunit beta
**NODE_2**	917179	G	G	G	G	C	C	C	C	C	C	C	C	C	C	C	missense_variant c.1413C>G p.Asp471Glu	rpoB	DNA-directed RNA polymerase subunit beta
**NODE_2**	917202	A	A	G	G	A	A	A	A	A	A	A	A	A	A	A	missense_variant c.1390T>C p.Ser464Pro	rpoB	DNA-directed RNA polymerase subunit beta
**NODE_2**	942440	C	C	C	C	C	C	C	C	C	C	C	T	C	C	C			
**NODE_2**	942452	A	A	A	A	A	A	A	A	A	A	A	G	A	A	A			
**NODE_2**	942485	T	T	T	T	T	T	T	T	T	T	T	C	T	T	T			
**NODE_3**	38470	T	T	T	T	T	T	T	T	T	T	T	T	T	T	C	synonymous_variant c.516T>C p.Arg172Arg	rlhA	23S rRNA 5-hydroxycytidine synthase
**NODE_3**	134055	C	C	C	C	C	C	C	C	T	C	C	C	C	C	C	stop_gained c.706C>T p.Gln236*	malL	Oligo-1,6-glucosidase

**NODE_3**	167055	T	T	T	T	A	A	T	T	T	T	A	A	T	A	A	missense_variant c.264T>A p.Phe88Leu	der_2	GTPase Der
**NODE_4**	153708	A	A	A	A	A	A	A	A	A	G	A	A	A	A	A	synonymous_variant c.969A>G p.Val323Val	pepS	Aminopeptidase PepS
**NODE_6**	18020	T	T	T	C	T	T	T	T	T	T	T	T	T	T	T	missense_variant c.361T>C p.Tyr121His		IS1182 family transposase ISSau3
**NODE_6**	46531	G	G	G	G	G	G	T	T	G	G	G	G	G	G	G	missense_variant c.409G>T p.Val137Phe		hypothetical protein
**NODE_9**	42869	T	T	T	T	T	T	C	C	C	C	T	T	C	T	T			

Abbr.: For numeration of isolates, see Supplementary Figure 1.

Supplementary Table 3: Accessory genome.

Gene	Annotation	No. of isolates	B 1	B 2	B 3	B 4	B 5	B 6	B 7	B 8	B 9	D 1	D 2	D 3	D 4	D 5	D 6
aceF	Dihydrolipoyllysine-residue acetyltransferase component of pyruvate dehydrogenase complex	1	0	0	0	0	0	0	0	0	0	0	0	1	0	0	0
alsT_1	Amino-acid carrier protein AlsT	1	0	0	0	0	0	0	0	0	0	0	0	0	0	0	1
clfA_1	Clumping factor A	1	0	1	0	0	0	0	0	0	0	0	0	0	0	0	0
clpP_2	ATP-dependent Clp protease proteolytic subunit	6	1	0	0	0	0	0	1	1	1	1	0	0	1	0	0
codY	GTP-sensing transcriptional pleiotropic repressor CodY	14	1	1	1	1	1	1	1	1	1	1	1	0	1	1	1
dltD_2	Protein DltD	1	0	1	0	0	0	0	0	0	0	0	0	0	0	0	0
est_2	Carboxylesterase	2	0	0	0	0	1	1	0	0	0	0	0	0	0	0	0
glpT_2	Glycerol-3-phosphate transporter	5	0	0	0	0	0	0	1	1	1	1	0	0	1	0	0
group_1	IS1182 family transposase ISSau3	8	0	1	0	0	1	1	1	0	0	0	1	1	1	1	0
group_100	hypothetical protein	6	1	0	0	0	0	0	1	1	1	1	0	0	1	0	0

group_101	hypothetical protein	6	1	0	0	0	0	0	1	1	1	1	0	0	1	0	0
group_102	hypothetical protein	6	1	0	0	0	0	0	1	1	1	1	0	0	1	0	0
group_103	hypothetical protein	6	1	0	0	0	0	0	1	1	1	1	0	0	1	0	0
group_104	hypothetical protein	6	1	0	0	0	0	0	1	1	1	1	0	0	1	0	0
group_105	hypothetical protein	6	1	0	0	0	0	0	1	1	1	1	0	0	1	0	0
group_106	hypothetical protein	6	1	0	0	0	0	0	1	1	1	1	0	0	1	0	0
group_108	hypothetical protein	6	1	0	0	0	0	0	1	1	1	1	0	0	1	0	0
group_109	hypothetical protein	6	1	0	0	0	0	0	1	1	1	1	0	0	1	0	0
group_110	hypothetical protein	6	1	0	0	0	0	0	1	1	1	1	0	0	1	0	0
group_111	hypothetical protein	6	1	0	0	0	0	0	1	1	1	1	0	0	1	0	0
group_112	hypothetical protein	6	1	0	0	0	0	0	1	1	1	1	0	0	1	0	0
group_113	hypothetical protein	6	1	0	0	0	0	0	1	1	1	1	0	0	1	0	0

group_114	hypothetical protein	6	1	0	0	0	0	0	1	1	1	1	0	0	1	0	0
group_115	hypothetical protein	6	1	0	0	0	0	0	1	1	1	1	0	0	1	0	0
group_116	hypothetical protein	6	1	0	0	0	0	0	1	1	1	1	0	0	1	0	0
group_117	hypothetical protein	6	1	0	0	0	0	0	1	1	1	1	0	0	1	0	0
group_118	hypothetical protein	6	1	0	0	0	0	0	1	1	1	1	0	0	1	0	0
group_119	hypothetical protein	6	1	0	0	0	0	0	1	1	1	1	0	0	1	0	0
group_120	hypothetical protein	6	1	0	0	0	0	0	1	1	1	1	0	0	1	0	0
group_121	hypothetical protein	6	1	0	0	0	0	0	1	1	1	1	0	0	1	0	0
group_122	hypothetical protein	6	1	0	0	0	0	0	1	1	1	1	0	0	1	0	0
group_123	hypothetical protein	6	1	0	0	0	0	0	1	1	1	1	0	0	1	0	0
group_125	hypothetical protein	6	1	0	0	0	0	0	1	1	1	1	0	0	1	0	0
group_126	hypothetical protein	6	1	0	0	0	0	0	1	1	1	1	0	0	1	0	0
group_127	hypothetical protein	6	1	0	0	0	0	0	1	1	1	1	0	0	1	0	0

group_128	hypothetical protein	6	1	0	0	0	0	0	1	1	1	1	0	0	1	0	0
group_129	hypothetical protein	6	1	0	0	0	0	0	1	1	1	1	0	0	1	0	0
group_130	hypothetical protein	6	1	0	0	0	0	0	1	1	1	1	0	0	1	0	0
group_131	hypothetical protein	6	1	0	0	0	0	0	1	1	1	1	0	0	1	0	0
group_132	hypothetical protein	6	1	0	0	0	0	0	1	1	1	1	0	0	1	0	0
group_133	hypothetical protein	6	1	0	0	0	0	0	1	1	1	1	0	0	1	0	0
group_134	hypothetical protein	6	1	0	0	0	0	0	1	1	1	1	0	0	1	0	0
group_135	hypothetical protein	6	1	0	0	0	0	0	1	1	1	1	0	0	1	0	0
group_136	hypothetical protein	6	1	0	0	0	0	0	1	1	1	1	0	0	1	0	0
group_137	hypothetical protein	6	1	0	0	0	0	0	1	1	1	1	0	0	1	0	0
group_138	hypothetical protein	6	1	0	0	0	0	0	1	1	1	1	0	0	1	0	0
group_139	hypothetical protein	6	1	0	0	0	0	0	1	1	1	1	0	0	1	0	0

group_140	hypothetical protein	6	1	0	0	0	0	0	1	1	1	1	0	0	1	0	0
group_141	hypothetical protein	6	1	0	0	0	0	0	1	1	1	1	0	0	1	0	0
group_143	putative autolysin PH	6	1	0	0	0	0	0	1	1	1	1	0	0	1	0	0
group_145	hypothetical protein	14	1	1	1	1	1	1	1	1	1	1	1	0	1	1	1
group_146	IS200/IS605 family transposase ISSep3	5	1	1	0	1	1	0	0	0	0	0	0	0	0	1	0
group_147	hypothetical protein	8	0	1	0	1	1	1	1	1	0	1	0	0	1	0	0
group_148	hypothetical protein	7	0	1	1	1	1	1	0	1	0	0	0	0	0	1	0
group_149	hypothetical protein	9	0	1	0	1	1	1	1	0	0	0	1	1	1	1	0
group_150	hypothetical protein	6	1	0	0	0	0	0	1	1	1	1	0	0	1	0	0
group_151	hypothetical protein	1	0	0	0	0	0	0	0	0	0	0	0	1	0	0	0
group_17	hypothetical protein	1	0	0	0	0	0	0	0	0	0	0	0	1	0	0	0
group_2	IS1182 family transposase ISSau3	1	0	0	0	0	0	0	0	0	0	0	0	0	0	1	0
group_21	hypothetical protein	1	0	1	0	0	0	0	0	0	0	0	0	0	0	0	0

group_22	putative sensor-like histidine kinase	12	1	0	0	0	1	1	1	1	1	1	1	1	1	1	1
group_23	putative sensor-like histidine kinase	8	0	1	1	1	0	0	1	1	1	1	0	0	1	0	0
group_2556	hypothetical protein	14	1	1	1	1	1	1	1	1	1	1	1	1	0	1	1
group_2557	hypothetical protein	3	0	0	0	0	0	0	1	0	0	0	1	0	0	0	1
group_3	IS1182 family transposase ISSau3	1	0	0	0	0	0	0	0	0	0	0	0	0	1	0	0
group_4	IS1182 family transposase ISSau3	2	0	0	0	0	0	1	1	0	0	0	0	0	0	0	0
group_5	IS1182 family transposase ISSau3	9	0	1	0	1	1	1	1	0	0	0	1	1	1	1	0
group_55	hypothetical protein	1	0	1	0	0	0	0	0	0	0	0	0	0	0	0	0
group_58	NAD-dependent malic enzyme	7	1	0	0	0	1	1	0	0	0	0	1	1	0	1	1
group_59	NAD-dependent malic enzyme	14	0	1	1	1	1	1	1	1	1	1	1	1	1	1	1
group_6	IS1182 family transposase ISSau3	9	0	1	0	1	1	1	1	0	0	0	1	1	1	1	0
group_7	IS1182 family transposase ISSau3	1	0	0	0	0	0	0	0	1	0	0	0	0	0	0	0

group_8	IS1182 family transposase ISSau3	1	0	0	0	0	0	0	0	1	0	0	0	0	0	0	0
group_83	hypothetical protein	14	1	1	1	1	1	1	1	1	1	1	1	1	0	1	1
group_84	hypothetical protein	14	1	1	1	1	1	1	1	1	1	1	1	1	0	1	1
group_85	hypothetical protein	14	1	1	1	1	1	1	1	1	1	1	1	1	0	1	1
group_87	hypothetical protein	6	1	0	0	0	0	0	1	1	1	1	0	0	1	0	0
group_88	hypothetical protein	6	1	0	0	0	0	0	1	1	1	1	0	0	1	0	0
group_89	hypothetical protein	6	1	0	0	0	0	0	1	1	1	1	0	0	1	0	0
group_9	IS1182 family transposase ISSau3	6	0	0	1	0	0	1	0	0	1	1	0	0	0	1	1
group_90	hypothetical protein	6	1	0	0	0	0	0	1	1	1	1	0	0	1	0	0
group_91	hypothetical protein	6	1	0	0	0	0	0	1	1	1	1	0	0	1	0	0
group_93	hypothetical protein	6	1	0	0	0	0	0	1	1	1	1	0	0	1	0	0
group_94	hypothetical protein	6	1	0	0	0	0	0	1	1	1	1	0	0	1	0	0
group_95	hypothetical protein	6	1	0	0	0	0	0	1	1	1	1	0	0	1	0	0

group_96	hypothetical protein	6	1	0	0	0	0	0	1	1	1	1	0	0	1	0	0
group_97	hypothetical protein	6	1	0	0	0	0	0	1	1	1	1	0	0	1	0	0
group_98	hypothetical protein	6	1	0	0	0	0	0	1	1	1	1	0	0	1	0	0
group_99	hypothetical protein	6	1	0	0	0	0	0	1	1	1	1	0	0	1	0	0
hlb_1	Phospholipase C	6	1	0	0	0	0	0	1	1	1	1	0	0	1	0	0
hpf_2	Ribosome hibernation promotion factor	4	0	0	0	0	0	0	1	1	1	1	0	0	0	0	0
hutH_1	Histidine ammonia-lyase	1	0	0	0	0	0	0	0	0	0	0	0	0	0	0	1
lexA_2	LexA repressor	6	1	0	0	0	0	0	1	1	1	1	0	0	1	0	0
malL	Oligo-1,6-glucosidase	14	1	1	1	1	1	1	1	1	1	1	0	1	1	1	1
malL_2	Oligo-1,6-glucosidase	2	0	0	0	0	0	0	0	0	1	0	1	0	0	0	0
malR	HTH-type transcriptional regulator MalR	2	0	0	0	0	0	0	0	0	1	0	1	0	0	0	0
malR	HTH-type transcriptional regulator MalR	14	1	1	1	1	1	1	1	1	0	1	1	1	1	1	1

mntA_2	Manganese-binding lipoprotein MntA	5	0	0	0	0	0	0	1	1	1	1	0	0	1	0	0
mprF	Phosphatidylglycerol lysyltransferase	14	1	1	1	1	1	1	1	1	1	1	1	1	1	1	0
mprF_2	Phosphatidylglycerol lysyltransferase	6	0	0	0	0	0	0	1	1	1	1	0	0	1	0	1
prkC_2	Serine/threonine-protein kinase PrkC	1	0	0	0	0	0	0	0	0	0	0	0	0	0	0	1
pycA_1	Pyruvate carboxylase	1	0	1	0	0	0	0	0	0	0	0	0	0	0	0	0
rsmH_1	Ribosomal RNA small subunit methyltransferase H	2	0	0	1	1	0	0	0	0	0	0	0	0	0	0	0
sak	Staphylokinase	6	1	0	0	0	0	0	1	1	1	1	0	0	1	0	0
scn_3	Staphylococcal complement inhibitor	6	1	0	0	0	0	0	1	1	1	1	0	0	1	0	0
spa_2	Immunoglobulin G-binding protein A	5	0	0	0	0	0	0	1	1	1	1	0	0	1	0	0
ssbA_2	Single-stranded DNA-binding protein A	6	1	0	0	0	0	0	1	1	1	1	0	0	1	0	0
thiI_1	putative tRNA sulfurtransferase	1	0	0	0	0	0	0	0	0	0	0	0	0	0	0	1
uhpT	Hexose-6-phosphate:phosphate antiporter	10	1	1	1	1	1	1	0	0	0	0	1	1	0	1	1
uhpT	Hexose-6-phosphate:phosphate antiporter	8	0	1	1	1	0	0	1	1	1	1	0	0	1	0	0

xerC_3	Tyrosine recombinase XerC	6	1	0	0	0	0	0	1	1	1	1	0	0	1	0	0
yciC_3	Putative metal chaperone YciC	2	0	0	1	1	0	0	0	0	0	0	0	0	0	0	0

Abbr.: For numeration of isolates, see Supplementary Figure 1. Absence of the respective gene is presented as 0, presence as 1.

Supplementary Table 4: Phenotypic AMR.

	IEC^{+}											
	**B1**		**B7**		**B8**		**B9**		**D1**		**D4**	
	MIC	Int	MIC	Int	MIC	In.	MIC	Int	MIC	Int	MIC	Int
**OXA**	≤0.25	S	≤0.25	S	0,5	S	1	S	0.5	S	≤0.25	S
**PEN**	≤0.03	S	≤0.03	S	≤0.03	S	0,12	S	≤0.03	S	≤0.03	S
**LVX**	≤0.12	S	0,25	S	0,5	S	0,5	S	0.25	S	0,5	S
**SXT**	≤10	S	≤10	S	≤10	S	≤10	S	≤10	S	≤10	S
**GEN**	≤0.5	S	≤0.5	S	≤0.5	S	≤0.5	S	≤0.5	S	≤0.5	S
**CLI**	0.25	S	0,25	S	0.25	S	0.25	S	0.25	S	0.25	S
**ERY**	1	S	0,5	S	0,5	S	0.5	S	≤0.25	S	≤0.25	S
**VAN**	1	S	≤0.5	S	1	S	≤0.5	S	≤0.5	S	≤0.5	S
**TEC**	2	S	≤0.5	S	1	S	≤0.5	S	≤0.5	S	1	S
**RIF**	**>32***	**R**	0,064*	S	0,064*	S	0,064*	S	0.125*	S	0,094*	S
**LZD**	2	S	4	S	4	S	2	S	4	S	2	S
**TET**	≤1	S	≤1	S	≤1	S	≤1	S	≤1	S	≤1	S
**TGC**	≤0.12	S	≤0.12	S	≤0.12	S	≤0.12	S	≤0.12	S	≤0.12	S
**FOF**	≤8	S	**≥128**	**R**	**≥128**	**R**	**≥128**	**R**	**≥128**	**R**	**≥128**	**R**
**FA**	≤0.5	S	≤0.5	S	≤0.5	S	≤0.5	S	≤0.5	S	≤0.5	S
**DAP**	0.25	S	≤0.12	S	≤0.12	S	≤0.12	S	≤0.12	S	≤0.12	S

	IEC^{-}																	
	**B2**		**B3**		**B4**		**B5**		**B6**		**D2**		**D3**		**D5**		**D6**	
	MIC	Int	MIC	Int	MIC	Int	MIC	Int	MIC	Int	MIC	Int	MIC	Int	MIC	Int	MIC	Int
**OXA**	≤0.25	S	0,5	S	0,5	S	1*	S	1*	S	0.5	S	**16***	**R**	**12***	**R**	1	S
**PEN**	≤0.03	S	0,12	S	0,12	S	0.06	S	0,12	S	≤0.03	S	0.25	S	0.25	S	0.12	S
**LVX**	0.5	S	1	S	1	S	0.25	S	1	S	0.5	S	0.25	S	0.25	S	0,25	S
**SXT**	≤10	S	≤10	S	≤10	S	≤10	S	≤10	S	≤10	S	≤10	S	≤10	S	≤10	S
**GEN**	≤0.5	S	≤0.5	S	≤0.5	S	≤0,5	S	≤0,5	S	≤0.5	S	≤0.5	S	≤0.5	S	≤0.5	S
**CLI**	0,25	S	≤0.12	S	≤0.12	S	≤0.12	S	≤0.12	S	0.25	S	≤0.12	S	≤0.12	S	0.25	S
**ERY**	1	S	0,5	S	1	S	0,5	S	≤0.25	S	≤0.25	S	0.5	S	0.5	S	0.5	S
**VAN**	≤0.5	S	≤0.5	S	≤0.5	S	≤0,5	S	≤0.5	S	≤0.5	S	≤0.5	S	≤0.5	S	≤0.5	S
**TEC**	≤0.5	S	2	S	≤0.5	S	≤0,5	S	≤0.5	S	1	S	≤0.5	S	≤0.5	S	≤0.5	S
**RIF**	0,012*	S	**16***	**R**	**6***	**R**	0,125*	S	0,25*	S	0,094*	S	0,19*	S	0,19*	S	0,19*	S
**LZD**	2	S	2	S	1	S	4	S	4	S	4	S	4	S	4	S	4	S
**TET**	≤1	S	≤1	S	≤1	S	2	S	≤1	S	≤1	S	≤1	S	≤1	S	≤1	S
**TGC**	≤0.12	S	≤0.12	S	≤0.12	S	≤0,12	S	≤0,12	S	≤0.12	S	≤0.12	S	≤0.12	S	≤0.12	S
**FOF**	**≥128**	**R**	**≥128**	**R**	**≥128**	**R**	**≥128**	**R**	**≥128**	**R**	**≥128**	**R**	**≥128**	**R**	**≥128**	**R**	**≥128**	**R**
**FA**	≤0.5	S	≤0.5	S	≤0.5	S	≤0.5	S	≤0.5	S	≤0.5	S	≤0.5	S	≤0.5	S	≤0.5	S
**DAP**	≤0.12	S	1	S	1	S	0.5	S	≤0.12	S	≤0.12	S	0.5	S	0.5	S	0.5	S

Abbr.: OXA oxacillin, PEN penicillin, LVX levofloxacin, SXT trimethoprim-sulfamethoxazole, GEN gentamicin, CLI clindamycin, ERY erythromycin, VAN vancomycin, TEC teicoplanin, RIF rifampicin, LZD linezolid, TET tetracycline, TGC tigecycline, FOF Fosfomycin, FA fusidic acid, DAP daptomycin, IEC immune evasion cluster, MIC minimal inhibitory concentration, Int interpretation. For enumeration of isolates, please see Supplementary Figure 1. *MIC determined by MIC test strip.

3 Supplementary Figures

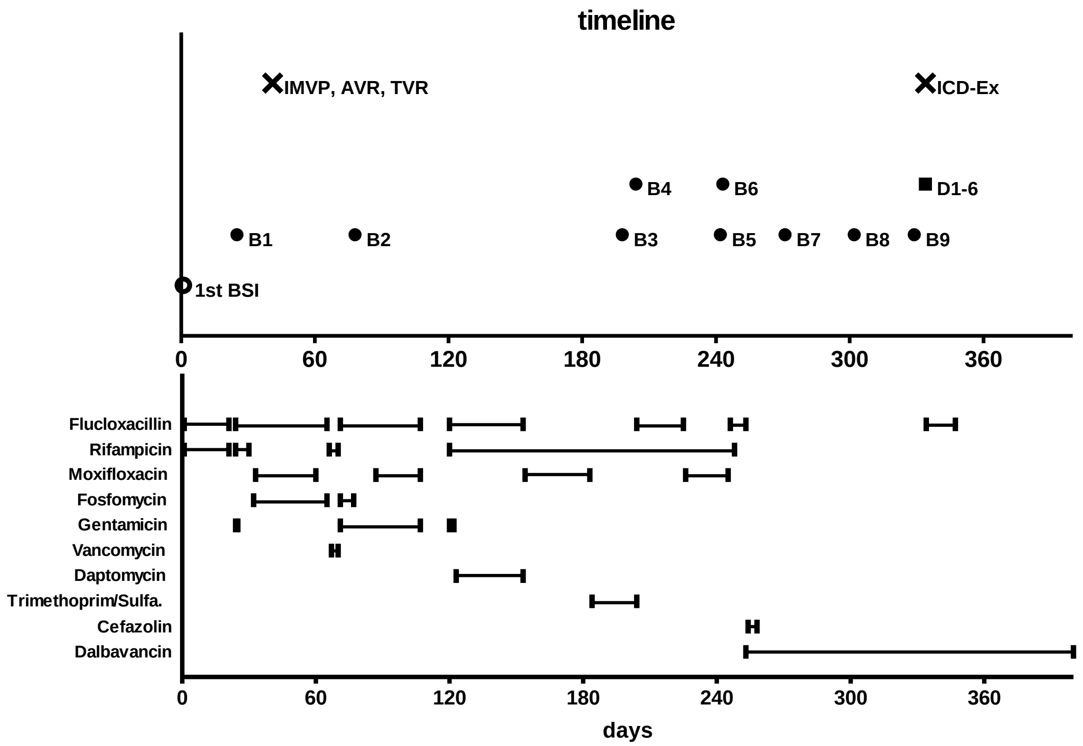

Supplementary Figure 1. Timeline of events and antibiotic therapy. Upper panel: Timeline of events. Day 0 is defined as the day of the first BSI episode (1st BSI). The *S. aureus* isolate of the first BSI was not available for analyses. B1-B9: Blood culture isolates of following BSI episodes. D1-D6: *S. aureus* isolates from the Device (implantable cardioverter-defibrillator, ICD). Abbr.: IMVP: Inspection of mitral valve prothesis; AVR: Aortic valve replacement; TVR: Tricuspidal valve replacement; ICD-Ex: ICD explantation. There were no episodes of BSI after explantation of the ICD. Lower panel: Overview over antimicrobial therapy. The timespan an antibiotic was given is visible as black line. Dosages: Flucloxacillin 2 g every 4 h or 3 g every 6 h; rifampicin 450 mg every 12 h or 600 mg every 24h; moxifloxacin 400 mg every 24 h; fosfomycin 4 g or 5 g every 24 h adjusted to current renal function; gentamicin 240 mg, 200 mg, 160 mg or 80 mg every 2 h according to drug monitoring results; vancomycin 1000mg on the first day, then 500mg according to drug monitoring results; daptomycin 500 mg or 700 mg every 24 h; trimethoprim/sulfamethoxazole 960 mg every 12h; cefazolin 2 g every 8 h; dalbavancin 1500 mg every 7 days.

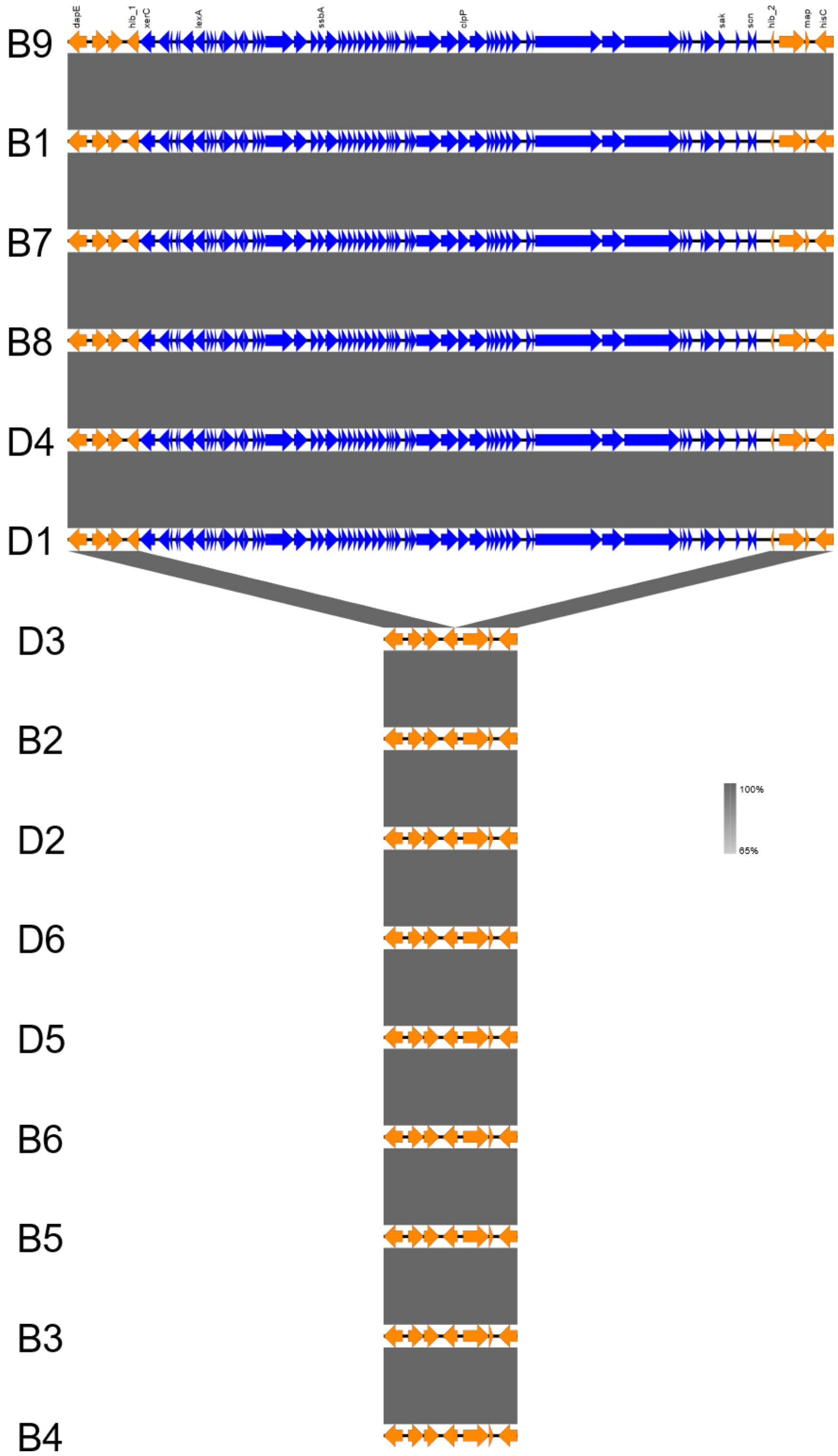

Supplementary Figure 2. Genetic context of the prophage inserted in the *hlb* gene. Abbr.: *dapE* probable succinyl-diaminopimelate desuccinylase; *hlb* hemolysin b; *xerC* tyrosine recombinase; *lexA* LexA repressor; *ssbA Staphylococcus aureus* single-stranded DNA-binding protein; *clpP* ATP-dependent Clp protease proteolytic subunit; *sak* staphylokinase; *scn* staphylococcal complement inhibitor; *map* Major histocompatibility complex class II analogue protein; *hisC* Histidinol-phosphate aminotransferase.

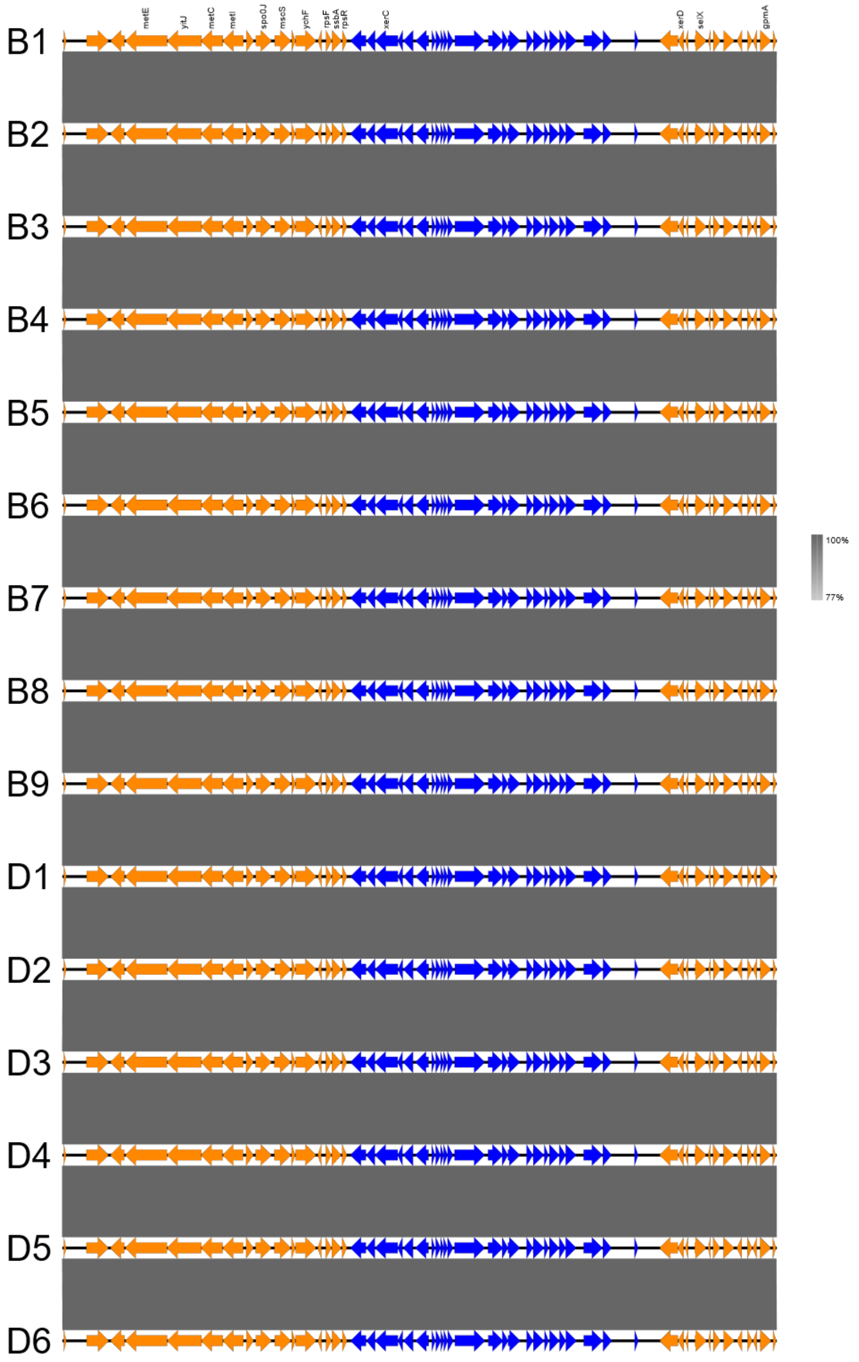

Supplementary Figure 3. Genetic context of the second mobile genetic element inserted after the gene *ychF*. Abbr.: *metE* 5-methyltetrahydropteroyltriglutamate-homocysteine methyltransferase; *yitJ* 5,10-methylenetetrahydrofolate reductase; *metC* aminotransferase class I/II-fold pyridoxal phosphate-dependent enzyme; *metI* Aminotransferase class I/II-fold pyridoxal phosphate-dependent enzyme; *spoOJ* SpoOJ protein; *mscS* mechanosensitive ion channel family protein; *ychF* ribosome-binding ATPase YchF; *rpsF* 30S ribosomal protein S6; *ssbA* single-stranded DNA-binding protein: *rpsR* 30S ribosomal protein S18; *xerC* Tyrosine recombinase XerC; *xerD* Tyrosine recombinase XerD; *selX* Enterotoxin-like toxin X; *gpmA* 2,3-bisphosphoglycerate-dependent phosphoglycerate mutase.

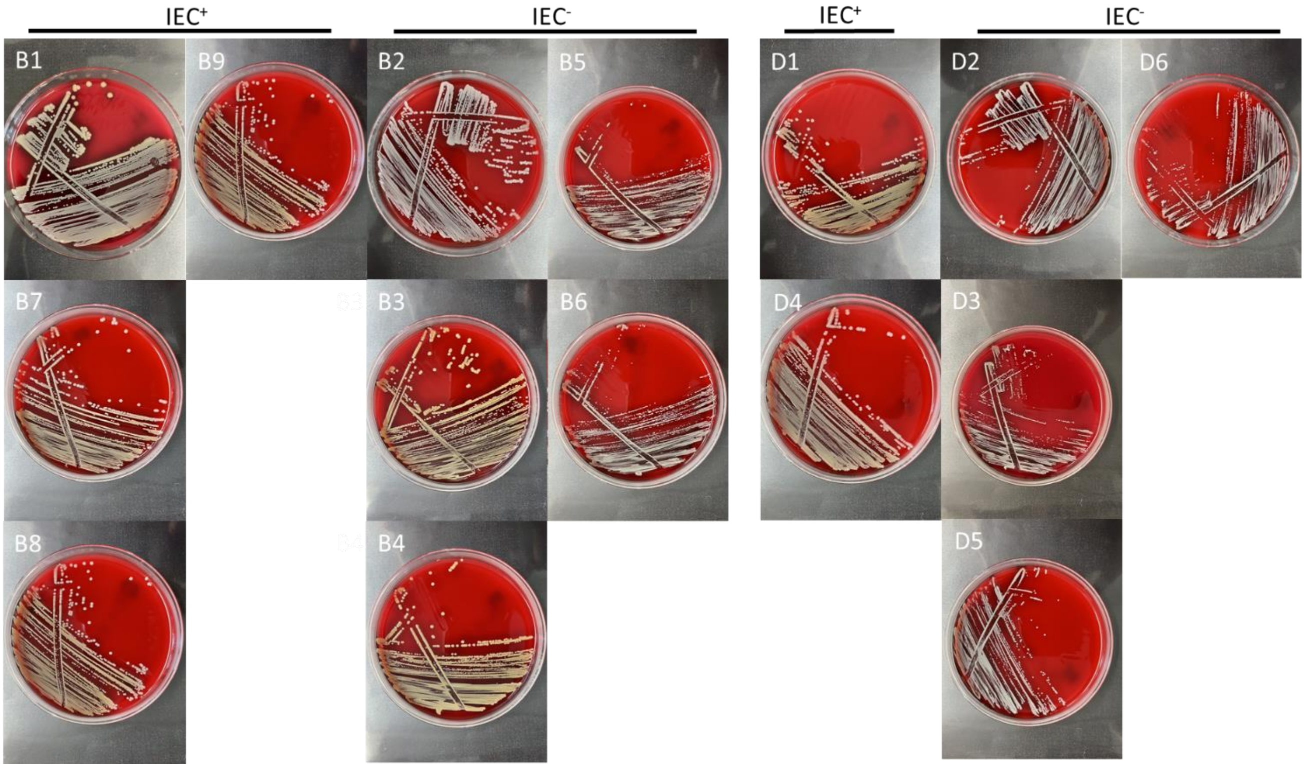

Supplementary Figure 4. Macromorphology of *S. aureus* isolates on Columbia blood agar. Morphology of isolates on Columbia blood agar following 24h cultivation. For numbering of isolates, see Supplementary Figure 1.

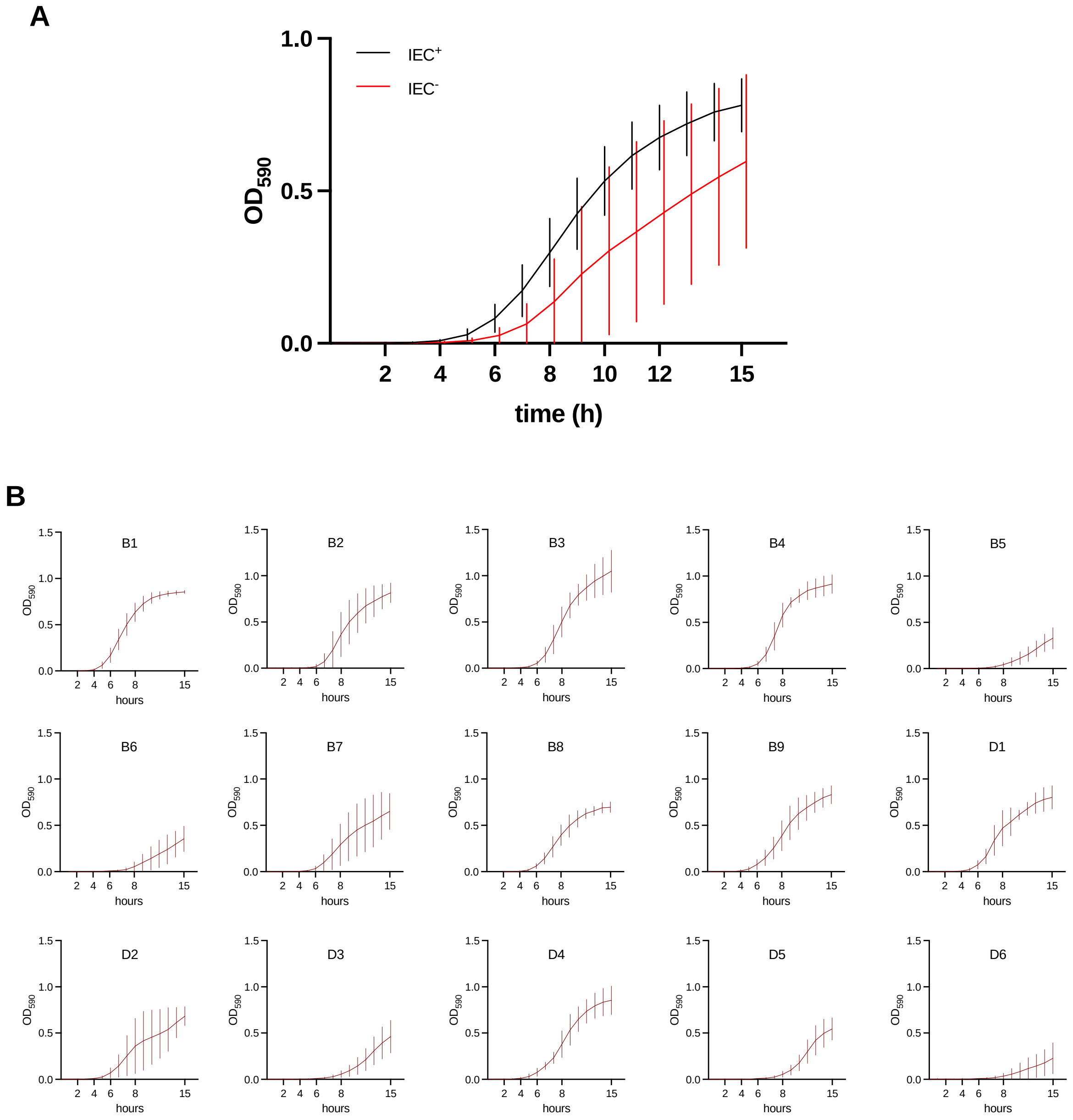

Supplementary Figure 5. Growth characteristics of bacterial isolates. Growth was determined by measuring the OD at 590nm every 5 min at 37°C for 15 hours in total. (A) There was no significant association between the growth characteristics of IEC$^-$ and IEC$^+$ isolates. Growth curve of all study isolates (performed as three independent experiments with technical triplicates each) in tryptic soy broth at 37° C, grouped by the IEC genotype. Data points of isolates belonging to the IEC$^-$ group were nudged on the x-axis by 10 data points for better visibility of standard deviations (only shown at full hours). (B) Individual growth curves of all study isolates (mean of three biological triplicates shown with standard deviation at full hours).

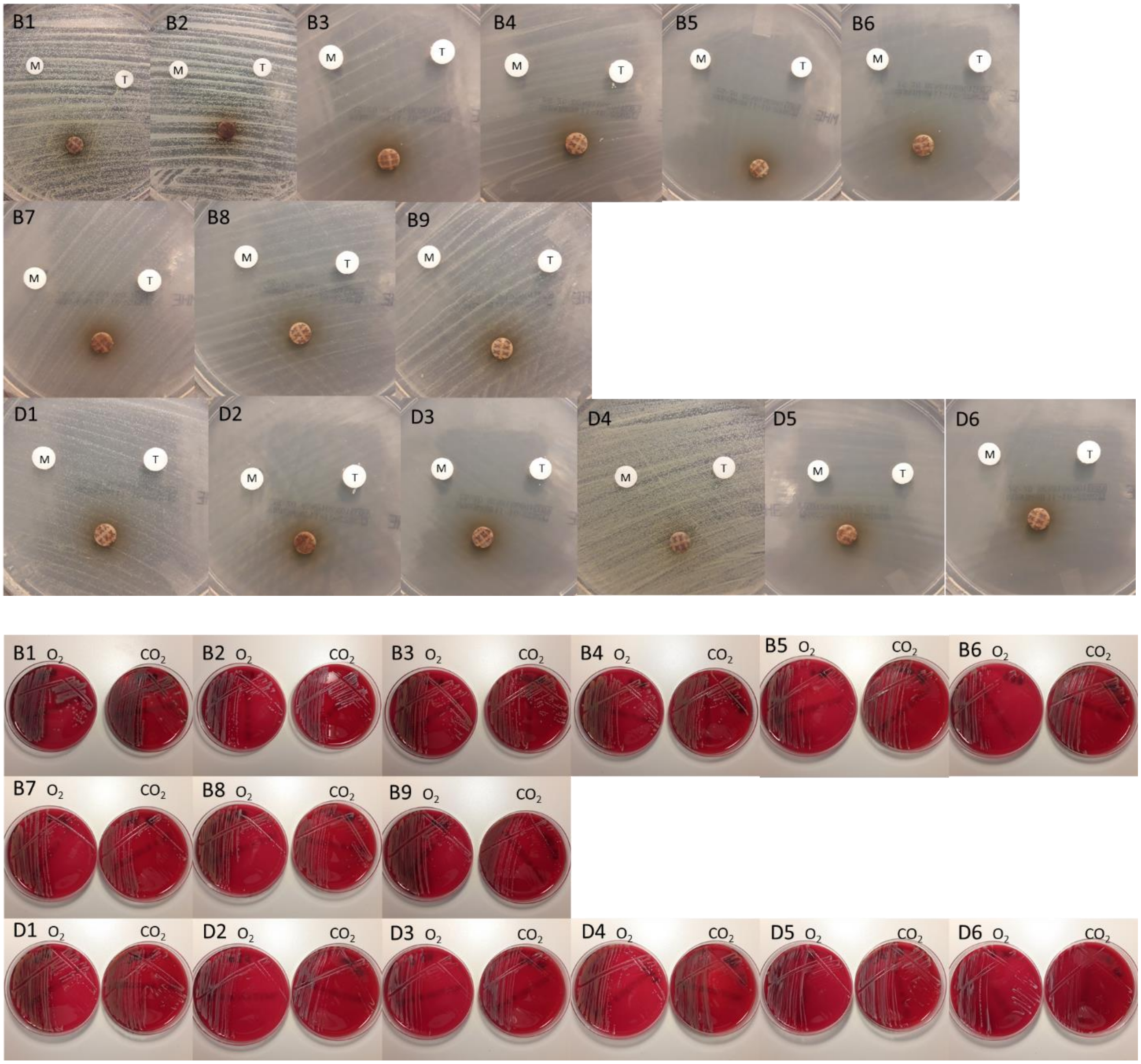

Supplementary Figure 6. Testing for auxotrophy. Upper panel: Testing for auxotrophy on Mueller-Hinton agar with impregnated discs with menadione (M), thymidine (T) and hemin (dark discs; X). Lower panel: Overnight culture on Columbia blood sheep agar with or without 5% CO_2. For enumeration of isolates, see Supplementary Figure 1.

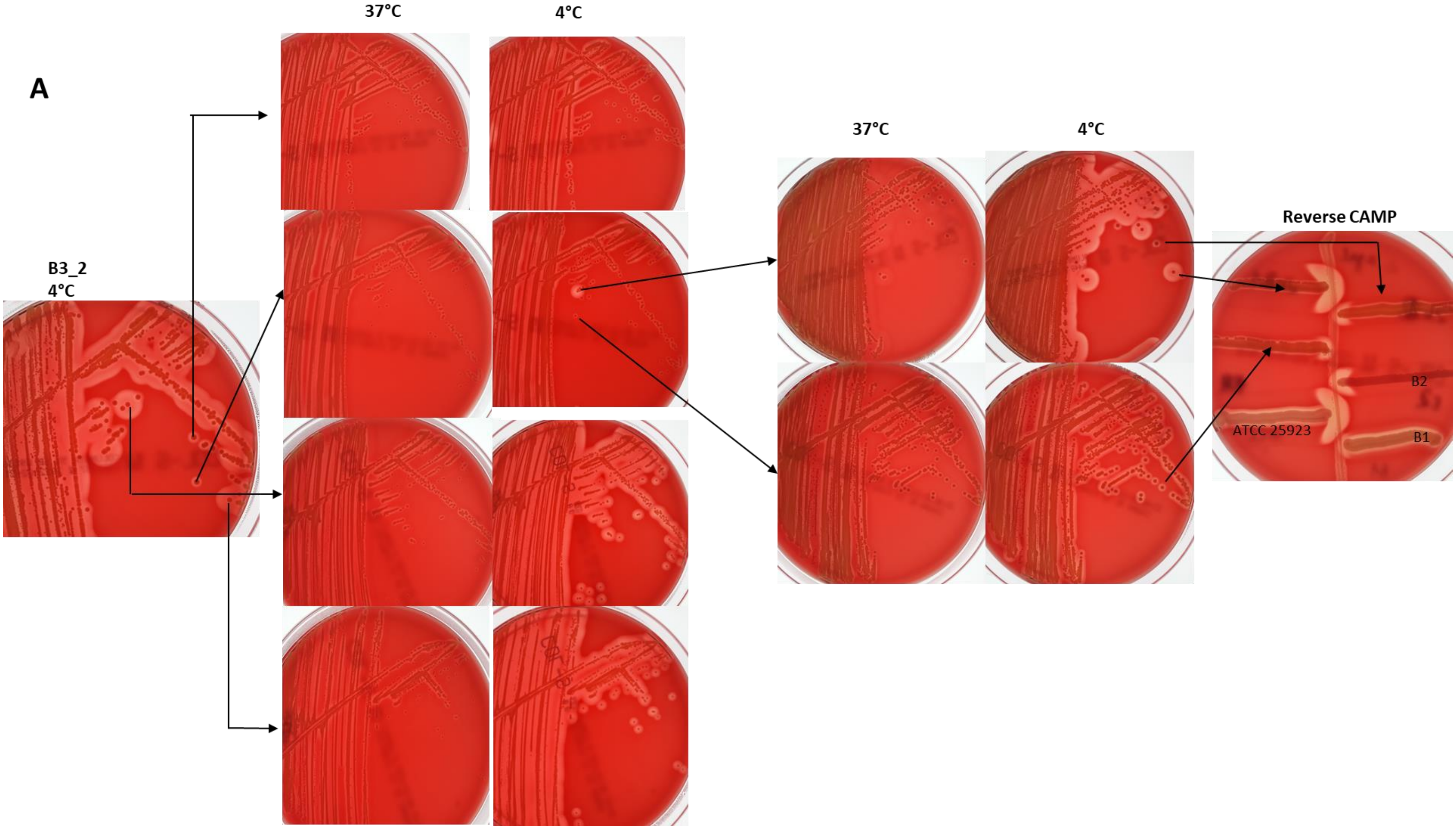
A
37°C
4°C
37°C
4°C
Reverse CAMP
B3_2
4°C
B2
ATCC 25923
B1

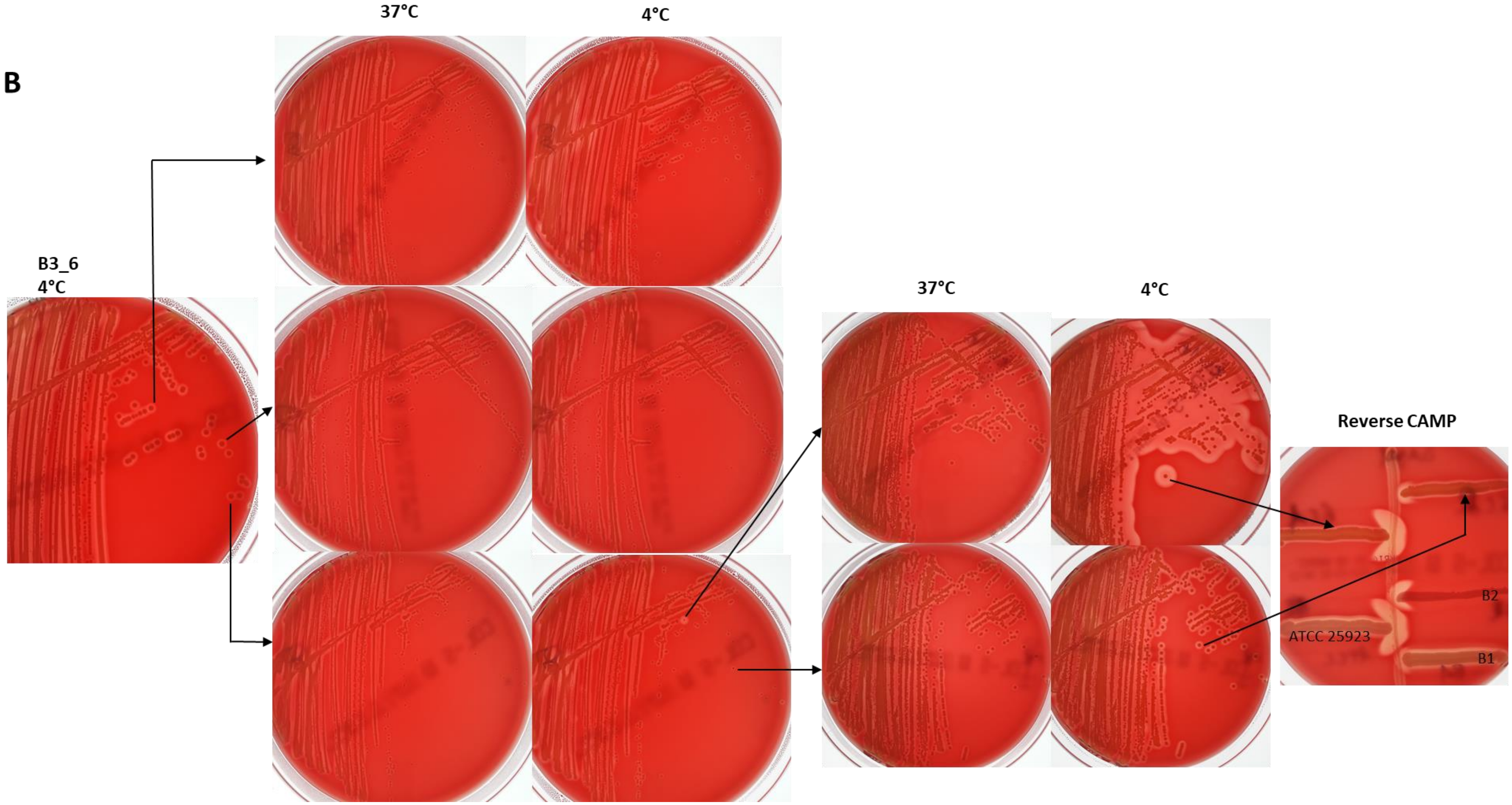

Supplementary Figure 7. Testing for β-hemolysis. Single colonies of isolate B3 were subcultured on 5% sheep blood agar at 37°C overnight, then imaged (named 37°C), incubated at 4°C overnight and then imaged again (named 4°C). Enlargement of the hemolysis zone following incubation for 4°C is indicating β-hemolysin production (hot-cold effect). For the reverse CAMP test, the tested colonies were streaked perpendicularly to a *Streptococcus agalactiae* ATCC 27956 reference strain in the middle of a 5% sheep blood agar plate. Enlargement of the hemolysis zone near to the *S. agalactiae*, so called "arrowhead", indicated β-hemolysin production. S. aureus ATCC 25923 was used as positive control and isolates B1 and B2 for comparison. (A) Subcultures of B3_2, (B) subcultures of B3_6.
